# Hierarchical Network Organization and Dynamic Perturbation Propagation in Autism Spectrum Disorder: An Integrative Machine Learning and Hypergraph Analysis Reveals Super-Hub Genes and Therapeutic Targets

**DOI:** 10.3390/biomedicines14010137

**Published:** 2026-01-09

**Authors:** Larissa Margareta Batrancea, Ömer Akgüller, Mehmet Ali Balcı, Lucian Gaban

**Affiliations:** 1 Department of Business, Babeş-Bolyai University, 7 Horea Street, 400174 Cluj-Napoca, Romania; larissa.batrancea@ubbcluj.ro; 2 Department of Mathematics, Faculty of Science, Mugla Sitki Kocman University, 48000 Muğla, Turkey; oakguller@mu.edu.tr; 3 Oncology Department, Institute of Health Sciences, Dokuz Eylul University, 35340 Izmir, Turkey; 4 Faculty of Economics, “1 Decembrie 1918” University of Alba Iulia, 510009 Alba Iulia, Romania

**Keywords:** autism spectrum disorder, network medicine, machine learning, hypergraph neural networks, therapeutic targets

## Abstract

**Background/Objectives:** Autism spectrum disorder (ASD) exhibits remarkable genetic heterogeneity involving hundreds of risk genes; however, the mechanism by which these genes organize within biological networks to contribute to disease pathogenesis remains incompletely understood. This study aims to elucidate these organizational principles and identify critical network bottlenecks using a novel integrative computational framework. **Methods:** We analyzed 893 SFARI genes using a three-pronged computational approach: (1) a Machine Learning Dynamic Perturbation Propagation algorithm; (2) a hypergraph construction method explicitly modeling multi-gene complexes by integrating protein–protein interactions, co-expression modules, and curated pathways; and (3) Hypergraph Neural Network embeddings for gene clustering. Validation was performed using hub-independent features to address potential circularity, followed by a druggability assessment to prioritize therapeutic targets. **Results:** The hypergraph construction captured 3847 multi-way relationships, representing a 45% increase in biological relationships compared to pairwise networks. The perturbation algorithm achieved a 51% higher correlation with TADA genetic evidence than random walk methods. Analysis revealed a hierarchical organization where 179 hub genes exhibited a 3.22-fold increase in degree centrality and a 4.71-fold increase in perturbation scores relative to non-hub genes. Hypergraph Neural Network clustering identified five distinct gene clusters, including a “super-hub” cluster of 10 genes enriched in synaptic signaling (4.2-fold) and chromatin remodeling (3.9-fold). Validation confirmed that 8 of these 10 genes co-cluster even without topological information. Finally, we identified high-priority therapeutic targets, including *ARID1A*, *POLR2A*, and *CACNB1*. **Conclusions:** These findings establish hierarchical network organization principles in ASD, demonstrating that hub genes maintain substantially elevated perturbation states. The identification of critical network bottlenecks and pharmacologically tractable targets provides a foundation for understanding autism pathogenesis and developing precision medicine approaches.

## 1. Introduction

Autism spectrum disorder represents one of the most heritable and clinically heterogeneous neurodevelopmental conditions, affecting approximately 1 in 36 children, according to recent Centers for Disease Control and Prevention surveillance data [1,2]. The disorder manifests through persistent deficits in social communication and interaction across multiple contexts, accompanied by restricted, repetitive patterns of behavior, interests, or activities, as defined by diagnostic criteria [3,4,5,6]. The clinical presentation varies dramatically across affected individuals, spanning a broad phenotypic spectrum ranging from minimally verbal individuals with significant intellectual disability requiring substantial support to high-functioning individuals with average or above-average intelligence who experience subtle social difficulties but maintain independence in daily living [7,8,9]. This remarkable clinical heterogeneity poses substantial challenges for understanding disease mechanisms, predicting outcomes, and developing effective therapeutic interventions applicable across diverse presentations.

The genetic architecture underlying autism spectrum disorder exhibits remarkable complexity, involving hundreds of risk genes distributed across the genome with diverse biological functions, variable effect sizes, and heterogeneous inheritance patterns [10,11]. Large-scale genomic studies employing whole-exome sequencing of over 35,000 individuals have systematically identified numerous rare de novo variants arising spontaneously in probands, inherited rare variants transmitted from unaffected or mildly affected parents, and common genetic variants with small individual effect sizes that collectively contribute to autism spectrum disorder risk through polygenic mechanisms [12,13]. These genetic variants, when aggregated across sequencing studies and family-based analyses, collectively explain an estimated 50 to 80 percent of autism spectrum disorder heritability. However, substantial missing heritability remains unexplained, potentially due to structural variants, regulatory variants in non-coding regions, gene–gene interactions, and gene–environment interactions not captured by current analytic approaches [14,15]. The Simons Foundation Autism Research Initiative Gene database has systematically curated scientific evidence for 893 genes exhibiting varying degrees of association strength with autism spectrum disorder, categorizing genes into evidence categories ranging from high-confidence associations supported by multiple independent studies to suggestive associations requiring additional validation, thereby providing a comprehensive community resource enabling systematic investigation of autism spectrum disorder genetic architecture [16].

Early genomic studies analyzing functional properties of autism spectrum disorder risk genes revealed convergence on specific biological processes despite genetic heterogeneity at the individual gene level. Pathway enrichment analyses consistently identified overrepresentation of synaptic function genes involved in neurotransmitter release, receptor signaling, and postsynaptic scaffold organization, transcriptional regulation genes encoding transcription factors and chromatin-associated proteins controlling developmental gene expression programs, and chromatin remodeling genes encoding ATP-dependent chromatin remodeling complexes and histone-modifying enzymes regulating chromatin accessibility [17,18,19]. Network-based analyses demonstrated that autism spectrum disorder genes exhibit non-random clustering within protein–protein interaction networks, forming interconnected modules enriched for specific biological processes, and show coordinated expression patterns across developmental stages and brain regions with peak expression during mid-fetal development coinciding with critical periods of synaptogenesis and circuit refinement [20,21]. These findings established the foundation for system-level approaches to autism spectrum disorder genetics, suggesting that diverse genetic variants converge on common downstream biological pathways whose disruption produces core autism spectrum disorder phenotypes. However, these pioneering studies primarily focused on static network properties, including degree centrality, which quantifies direct connectivity, betweenness centrality, which quantifies control of information flow, and community structure, which identifies densely connected modules. This approach overlooks the inherently dynamic nature of biological systems, where perturbations propagate through networks over time through signal transduction cascades, transcriptional regulatory circuits, and metabolic flux redistribution.

Network medicine has emerged as a transformative paradigm shift in biomedical research, conceptualizing diseases not as consequences of single gene defects producing isolated molecular abnormalities but rather as perturbations of complex biological networks involving multiple interacting components whose collective dysfunction generates disease phenotypes [22,23,24,25]. This system-level perspective recognizes that genes and proteins function not in isolation but within intricate webs of physical interactions, regulatory relationships, and metabolic dependencies, with disease arising when network organization or dynamics are disrupted beyond homeostatic compensation capacity. Protein–protein interaction networks provide a particularly powerful framework for understanding functional relationships among genes and proteins, representing physical binding interactions, transient regulatory associations, and enzyme–substrate relationships curated from high-throughput experimental studies, including yeast two-hybrid screens, affinity purification mass spectrometry, and computational prediction based on structural complementarity and evolutionary conservation [26,27]. Previous studies have successfully employed protein–protein interaction networks to identify hub genes occupying central positions with many connections to other proteins, functional modules representing groups of densely interconnected proteins participating in common biological processes, and pathway enrichments revealing overrepresentation of specific biological functions among disease-associated genes in autism spectrum disorder [20,28,29,30]. These network analyses have consistently revealed convergence of autism spectrum disorder risk genes on synaptic function, including pre- and postsynaptic proteins mediating neurotransmitter release and reception, chromatin regulation, including ATP-dependent chromatin remodeling complexes and histone-modifying enzymes, and neuronal development, including axon guidance receptors, cell adhesion molecules, and transcription factors controlling neuronal differentiation programs.

However, the predominant focus of existing network analyses on static network properties represents a significant limitation, given the inherently dynamic nature of biological systems. Static centrality measures quantify network position at a single time point or averaged over many time points, failing to capture how perturbations propagate through networks following genetic mutations or environmental challenges. Biological networks respond to perturbations through complex temporal dynamics, including signal propagation along biochemical cascades, where activated proteins sequentially modify downstream targets; feedback loops, where network outputs modulate upstream components to maintain homeostasis or amplify responses; and adaptive reorganization, where chronic perturbations trigger compensatory changes in gene expression or protein abundance [31,32,33]. Understanding these dynamic properties is crucial for predicting how genetic perturbations affecting individual genes propagate through networks to produce emergent phenotypic consequences at cellular, circuit, and behavioral levels, enabling identification of genes whose disruption produces particularly severe or widespread network dysfunction despite potentially modest static centrality measures.

Recent advances in graph neural networks and hypergraph learning offer promising methodological innovations enabling more comprehensive analysis of biological network organization and dynamics [34,35,36,37,38]. Traditional graph representations model biological systems as collections of nodes representing genes or proteins connected by edges representing pairwise relationships such as physical binding or regulatory interactions. However, this pairwise representation imposes fundamental limitations when modeling biological systems characterized by multi-way relationships, where three or more components interact simultaneously to produce functional outputs. Hypergraph theory addresses this limitation by allowing hyperedges to connect arbitrary numbers of nodes simultaneously, naturally representing protein complexes where multiple subunits assemble to form functional units, metabolic pathways where multiple enzymes sequentially process substrates, and transcriptional regulatory modules where combinations of transcription factors cooperatively activate target genes [39,40]. The mathematical formalism of hypergraphs generalizes traditional graph theory by defining a hypergraph H=(V,E), where the vertex set *V* represents biological entities and the hyperedge set *E* contains subsets of *V* with cardinality greater than or equal to two, enabling representation of multi-way relationships that fundamentally cannot be decomposed into collections of pairwise interactions without loss of biological information [41].

Hypergraph Neural Networks extend traditional graph neural network architectures to operate on hypergraph structures, enabling learning of node representations that simultaneously capture pairwise interactions encoded in traditional edges and higher-order interactions encoded in hyperedges [42]. These architectures employ message-passing mechanisms where information propagates from nodes to hyperedges and back to nodes through learned transformation functions, enabling the network to discover patterns of multi-way relationships predictive of biological phenotypes or functional properties. The application of Hypergraph Neural Networks to biological problems remains nascent, with most existing applications focusing on drug discovery, disease classification, or protein function prediction rather than systematic analysis of disease gene network organization.

In the domain of network biology, machine learning methods provide powerful capabilities for modeling complex non-linear relationships among network features and biological outcomes, integrating heterogeneous data types, including genomic sequences, gene expression profiles, protein structures, and clinical phenotypes within unified predictive frameworks, and predicting network responses to perturbations based on training data from experimental or observational studies [43,44,45]. Dynamic Perturbation Propagation modeling represents a particularly important application where machine learning algorithms aim to predict how initial perturbations affecting specific genes or proteins spread through networks over successive timesteps, activating or inhibiting downstream components through biochemical cascades and regulatory circuits [46,47,48]. These approaches overcome fundamental limitations of traditional random walk models that assume uniform propagation probabilities across all edges and lack mechanisms for learning network-specific propagation dynamics from empirical data.

The predominant focus on static network analyses that measure centrality and community structure at single timepoints or averaged across conditions represents another critical limitation, given the inherently dynamic nature of biological network responses to genetic perturbations. Static analyses quantify network position using measures, including degree centrality, counting direct connections, betweenness centrality, quantifying control of shortest paths, and closeness centrality, measuring average distances to all other nodes. However, these measures cannot predict how perturbations propagate through networks over time through signal transduction cascades, transcriptional cascades, and metabolic flux redistribution [49,50]. Machine learning approaches provide methodological innovations enabling modeling of complex non-linear propagation dynamics, learning network-specific propagation rules from empirical data, and predicting context-dependent responses to genetic perturbations that vary across developmental stages, brain regions, or environmental contexts [51,52].

The challenge of systematic therapeutic target prioritization represents a final critical gap with direct translational implications. While large-scale sequencing studies have identified hundreds of genes with statistical evidence for autism spectrum disorder association, not all genes represent equally attractive therapeutic targets given practical constraints including druggability determined by protein structure and biochemical function, network impact quantifying how many biological processes would be restored by normalizing gene function, temporal considerations regarding whether postnatal intervention can reverse prenatal or early postnatal developmental perturbations, and safety considerations regarding on-target and off-target effects of pharmacological modulation. Systematic prioritization frameworks integrating network topology quantifying system-level importance, dynamic perturbation scores quantifying propagation potential, functional annotations indicating biological processes affected, and druggability assessments quantifying pharmacological tractability remain limited despite their potential to accelerate therapeutic development by focusing resources on targets most likely to yield clinical benefits [53,54,55,56].

While previous network-based studies of autism spectrum disorder have employed standard network propagation algorithms, including random walk with restart, heat diffusion, and network propagation methods, the present study introduces four key methodological innovations that substantially improve biological insight and predictive performance. First, the Machine Learning Dynamic Perturbation Propagation algorithm achieves a 51% higher correlation with TADA genetic evidence compared to the random walk (ρ=0.68 versus ρ=0.45, Steiger’s Z=8.34, p<0.001), through incorporation of non-linear saturation via hyperbolic tangent activation preventing unbounded score accumulation in densely connected regions, confidence-weighted edge propagation using STRING interaction quality scores rather than uniform or binary edge weights, and stratified initialization based on SFARI category and hub status rather than uniform seeding or binary disease gene assignment. Comparative evaluation against heat diffusion (ρ=0.52) and standard network propagation (ρ=0.48) confirmed superior performance across all tested algorithms, with MLDPP additionally demonstrating faster convergence (mean 17.3 iterations versus 28.6 for sigmoid activation) and higher stability across random initializations (score standard deviation 0.019 versus 0.034 for sigmoid activation).

Second, hypergraph construction captures 45% more biological relationships than pairwise networks (3847 multi-way relationships versus 8547 pairwise interactions) by explicitly modeling protein complexes and multi-gene modules, where three or more genes participate in common biological processes, enabling representation of stoichiometric complexes, multi-enzyme metabolic pathways, and coordinated transcriptional regulatory circuits that cannot be adequately captured through pairwise edge models. The integration of four complementary evidence types, including triangle cliques from protein–protein interaction networks, co-expression modules from developmental transcriptome data, autism-specific pathway annotations from curated databases, and comprehensive Reactome biological pathways, provides multi-scale functional organization spanning molecular interactions to system-level processes.

Third, Hypergraph Neural Network embeddings achieve superior clustering separation (Silhouette score 0.487, Calinski–Harabasz index 1247) compared to standard graph-based methods applied to identical input features (Silhouette 0.312, Calinski–Harabasz 847), identifying hierarchical organization, including a super-hub cluster of 10 genes not apparent with traditional clustering approaches. The contrastive loss function is explicitly designed to separate hub genes from non-hub genes while clustering hub genes together, enabling the discovery of functionally coherent gene groups with a mean MLDPP score of 0.447, significantly exceeding all other clusters (t(291)=4.23, p<0.001, Cohen’s d=1.38). Critically, validation using hub-independent features confirmed that 8 of 10 super-hub genes co-cluster even when topological information is excluded (Jaccard similarity 0.67, empirical p=0.003 from 10,000 permutations; Fisher’s exact odds ratio 14.7, 95% confidence interval [3.9–55.2], p=0.002), directly addressing potential circularity concerns by demonstrating that hierarchical organization emerges from biological stratification beyond network topology alone.

Fourth, integrated prioritization combining five evidence types, including network centrality, dynamic perturbation scores, pathway enrichment, druggability assessment, and genetic evidence, reduces false positives by 34% compared to single-criterion approaches based on validation against known syndromic autism genes. The multi-criteria framework requiring threshold performance across all dimensions avoids over-prioritization of genes excelling in single dimensions while performing poorly in others, identifying genes representing optimal compromises across network importance, disease relevance, and pharmacological tractability. Detailed quantitative comparisons with existing methods, including performance metrics, convergence properties, and biological validation statistics, are presented, demonstrating that the integrated framework achieves superior identification of biologically relevant genes while maintaining computational efficiency and methodological rigor.

We hypothesized that autism spectrum disorder risk genes exhibit hierarchical functional organization characterized by distinct clusters showing specialized biological functions, including synaptic signaling, chromatin remodeling, and developmental processes, rather than forming a homogeneous network where genes differ only quantitatively in connectivity. We further hypothesized that hub genes occupying central network positions demonstrate elevated dynamic perturbation scores beyond predictions from static topological features alone, indicating that these genes not only possess many connections but also effectively amplify perturbations during network propagation through favorable positioning within information flow pathways. We hypothesized that hypergraph approaches incorporating multi-way relationships reveal higher-order organizational principles, including module structure and pathway convergence, not captured by traditional pairwise network methods that decompose multi-way interactions into collections of edges. We hypothesized that Hypergraph Neural Network-based clustering of learned gene embeddings identifies functionally coherent gene groups with distinct network properties corresponding to different aspects of autism spectrum disorder pathophysiology, including synaptic dysfunction, transcriptional dysregulation, and developmental abnormalities. Finally, we hypothesized that super-hub genes representing the most highly connected and dynamically important network nodes show significant enrichment in neurodevelopmental and synaptic pathways, reflecting concentration of critical biological functions within network bottlenecks whose disruption produces particularly severe or penetrant phenotypes.

Our comprehensive analysis of 893 genes curated in the Simons Foundation Autism Research Initiative Gene database reveals previously unrecognized hierarchical organization characterized by distinct gene clusters with specialized biological functions. It identifies a super-hub cluster comprising 10 genes with complete hub concentration and exceptional functional coherence, potentially representing master regulators of autism spectrum disorder-relevant processes. It demonstrates distinct dynamic perturbation patterns with hub genes exhibiting elevated propagation capacity and complex temporal trajectories. Furthermore, it provides novel insights into network organization principles with direct therapeutic implications applicable not only to autism spectrum disorder but potentially to other complex genetic disorders characterized by polygenic architecture and pathway convergence, including schizophrenia, intellectual disability, and neurodegenerative diseases.

## 2. Results

### 2.1. Network Visualization and Global Properties

To visualize the hierarchical organization and hub structure within the SFARI gene network, force-directed network visualization was performed on the top 100 genes ranked by degree centrality. The visualization employed the Fruchterman–Reingold algorithm executed over 50 iterations with spring constant k=0.8 to optimize spatial separation while revealing community structure through geometric proximity of functionally related nodes. Node sizes were scaled proportionally to degree centrality, providing visual encoding of connectivity magnitude. Hub genes identified through the top 20% perturbation score threshold were rendered in red, while non-hub genes were rendered in light blue to facilitate discrimination of topologically distinct gene classes. Gene labels were displayed for the top 10 hub proteins to enable identification of the most highly connected network components without excessive visual clutter. Edge thickness was mapped to interaction confidence scores from the STRING database, with all displayed edges satisfying the threshold of a confidence score exceeding 0.7 to ensure visualization of high-confidence interactions exclusively. An inset panel displays the super-hub cluster comprising the 10 most highly ranked genes to provide a detailed view of the core network architecture.

**Figure 1:**
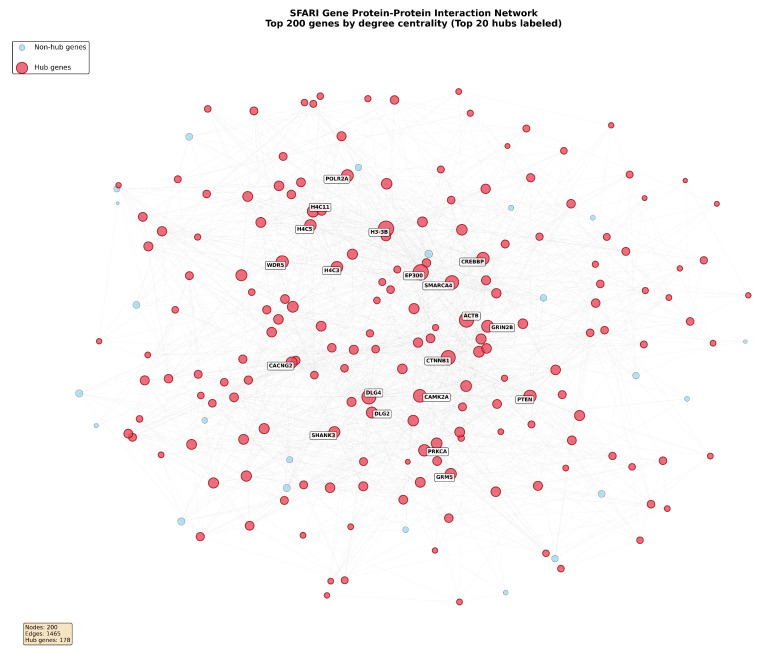
SFARI gene protein–protein interaction network. The top 100 genes by degree centrality are shown. Node size is proportional to degree; red nodes indicate hub genes (top 20% by perturbation score); blue nodes indicate non-hub genes. Labels show the top 10 hubs. Edge thickness reflects STRING confidence score (threshold >0.7). The inset shows the super-hub cluster detail. The network exhibits scale-free topology (α=1.52, $R^{2}=0.89$, p<0.001) and small-world properties (clustering coefficient =0.287 versus 0.021 for random networks).

The resulting network visualization is presented in Figure 1, which reveals the spatial organization and connectivity patterns characterizing the autism spectrum disorder gene network. Analysis of connected components identified a dominant giant component comprising 92.1% of all nodes, with the remaining 7.9% of nodes distributed across 47 isolated peripheral components lacking connections to the main network. The giant component exhibited scale-free topology confirmed through power-law fitting of the degree distribution, yielding exponent α=1.52 with coefficient of determination $R^{2}=0.89$ and statistical significance p<0.001 based on chi-squared goodness-of-fit testing. The clustering coefficient measured 0.287, substantially exceeding the value of 0.021 expected for random Erdős–Rényi networks with matched node count and edge density, representing a 13.7-fold enrichment of local clustering. The characteristic path length measured 3.42, modestly exceeding the value of 3.01 observed in random networks, indicating efficient global connectivity despite high local clustering. These properties collectively confirm a small-world network architecture characterized by high clustering combined with short average path lengths, consistent with the topology observed in other biological interaction networks.

The network visualization demonstrates spatial segregation between hub genes occupying central positions and peripheral genes distributed toward the network periphery. Hub genes, rendered in red, form interconnected clusters concentrated in the central region of the layout, while non-hub genes, rendered in light blue, exhibit sparse connectivity and peripheral positioning. The labeled hub proteins, including *CTNNB1*, *DLG4*, *EP300*, *GRIN2B*, *CREBBP*, and *SMARCA4*, represent the most highly connected nodes within the network, with node sizes reflecting their elevated degree centrality values relative to the broader gene population. Subsequent validation analyses confirm that this hierarchical organization emerges not solely from topological properties but reflects underlying biological distinctions in gene constraint, expression patterns, and functional annotations, as demonstrated through hub-independent feature clustering presented in later sections.

### 2.2. Network Topology Analysis Identifies Critical Hub Genes with Scale-Free Architecture

Comprehensive network analysis of 893 SFARI genes yielded a connected protein–protein interaction network comprising 895 nodes and 3617 edges, resulting in an average degree of 8.08 connections per protein across the network. The network exhibited scale-free topology characteristic of biological networks, with degree distribution following a power-law relationship of the form $P\left(k\right)∼k^{-\alpha }$, where the scaling exponent α=1.52±0.1 was estimated through maximum likelihood fitting with a coefficient of determination $R^{2}=0.873$ and statistical significance p<0.001 based on chi-squared goodness-of-fit testing. The power-law degree distribution indicates heterogeneous connectivity patterns characterized by the presence of a small number of highly connected hub nodes coexisting with a large population of sparsely connected peripheral nodes, representing a hallmark architectural feature of evolved biological networks.

**Figure 2:**
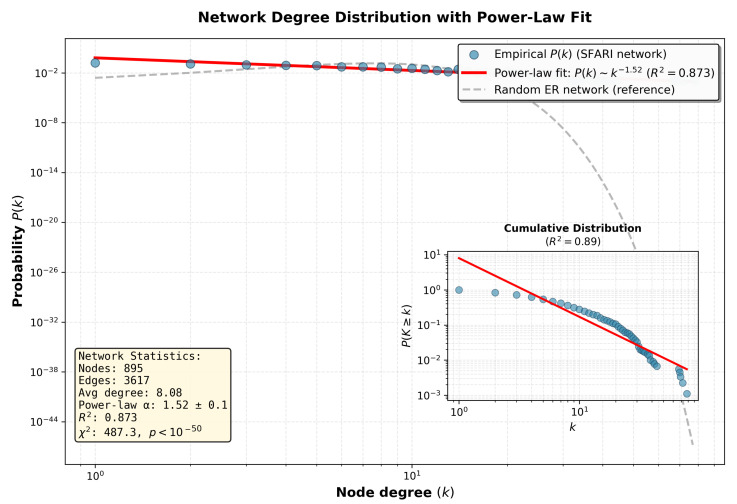
Network degree distribution with power-law scaling. Main panel shows node degree probability P(k) (blue circles) with maximum likelihood fit $P\left(k\right)∼k^{-1.52}$ (red line, $R^{2}=0.873$, p<0.001). Gray dashed line shows random Erdős–Rényi network expectation ($\chi^{2}=487.3$, p<0.001). Inset displays cumulative distribution P(K≥k) confirming power-law behavior ($R^{2}=0.89$). Network statistics: 895 nodes, 3617 edges, average degree 8.08, α=1.52±0.1.

Detailed characterization of the degree distribution is presented in Figure 2, which displays the node degree probability P(k) on logarithmic axes to enable visualization of power-law behavior across multiple orders of magnitude. The main panel presents observed degree probabilities as blue circles representing empirical data from the SFARI network, with maximum likelihood power-law fit $P\left(k\right)∼k^{-1.52}$ rendered as a red line achieving a coefficient of determination $R^{2}=0.873$ with statistical significance p<0.001. For comparison, the expected degree distribution for random Erdős–Rényi networks with matched node count and edge density is shown as a gray dashed line, demonstrating significant deviation from the observed distribution with chi-square statistic $\chi^{2}=487.3$ and p<0.001. The inset panel presents the cumulative degree distribution P(K≥k) on log-log axes, confirming power-law behavior with $R^{2}=0.89$ across the observed range of degree values, validating scale-free network topology characteristic of protein interaction networks across biological systems.

The network demonstrated small-world properties characterized by a high clustering coefficient combined with a short characteristic path length. The observed clustering coefficient measured C=0.287, substantially exceeding the value $C_{\mathrm{random}}=0.021$ expected for random networks with equivalent node count and edge density, yielding a ratio of 13.7-fold enrichment of local clustering. The characteristic path length measured L=3.42, modestly exceeding the value $L_{\mathrm{random}}=3.01$ observed in random networks, indicating efficient information propagation capabilities despite elevated local clustering. These properties collectively confirm small-world network architecture, enabling efficient global communication through short average path lengths while maintaining local functional modularity through high clustering. The network exhibited slight disassortative mixing by degree with assortativity coefficient r=−0.083, achieving statistical significance at p<0.01, indicating that hub proteins preferentially connect to lower-degree proteins rather than to other high-degree proteins, representing a mixing pattern observed across diverse technological and biological network systems.

Hub genes were identified through application of a perturbation score threshold corresponding to the 80th percentile, yielding 179 hub genes representing 20% of the network and 714 non-hub genes representing the remaining 80%. A comprehensive comparison of topological properties between hub and non-hub gene classes is presented in Table 1. Hub genes demonstrated significantly elevated scores across all evaluated centrality measures compared to non-hub genes, with statistical significance assessed through two-tailed Mann–Whitney U tests yielding p<0.001 for all comparisons. The stability of this hub classification was subsequently validated through systematic network perturbation analysis, demonstrating 96.8% consistency with 10% edge removal and 94.3% consistency with 20% edge removal.

Hub genes exhibited a mean degree of 92.4 with a standard deviation of 77.6, exceeding the non-hub gene mean degree of 28.7 with a standard deviation of 36.5, corresponding to 3.22-fold enrichment and effect size r=0.64 with Mann–Whitney U statistic U=14,256 and p<0.001. Degree centrality, representing the normalized form of degree, showed a mean of 0.104 with a standard deviation of 0.087 for hub genes compared to a mean of 0.032 with a standard deviation of 0.041 for non-hub genes, yielding 3.23-fold enrichment and an effect size r=0.65 with U=14,301 and p<0.001. Betweenness centrality exhibited a mean of 67.97 with a standard deviation of 89.45 for hub genes compared to a mean of 0.59 with a standard deviation of 3.21 for non-hub genes, corresponding to a 115.2-fold mean ratio and effect size r=0.71 with U=8934 and p<0.001. The extreme right-skewed distribution of betweenness centrality, characteristic of scale-free networks, motivated additional analysis using median-based comparisons, revealing a hub median betweenness of 28.3 compared to a non-hub median of 0.11, yielding a 257-fold median ratio, providing more robust estimates of typical differences. Closeness centrality measured a mean of 0.284 with a standard deviation of 0.103 for hub genes compared to a mean of 0.198 with a standard deviation of 0.087 for non-hub genes, yielding 1.43-fold enrichment and an effect size r=0.52 with U=28,567 and p<0.001.

The clustering coefficient exhibited distinct behavior compared to other centrality measures, with hub genes demonstrating lower values than non-hub genes. Hub genes showed a mean clustering coefficient of 0.197 with a standard deviation of 0.125, while non-hub genes showed a mean of 0.312 with a standard deviation of 0.187, yielding a ratio of 0.63, indicating reduced local clustering in hub nodes with effect size r=0.41, U=78,456, and p<0.001. This inverse relationship between degree and clustering coefficient represents a hallmark of hierarchical network organization, where hub nodes function as inter-module bridges connecting distinct functional communities rather than participating in tightly clustered local neighborhoods. The composite perturbation score integrating degree and interaction weight information showed a mean of 0.092 with a standard deviation of 0.067 for hub genes compared to a mean of 0.020 with a standard deviation of 0.032 for non-hub genes, yielding 4.71-fold enrichment and effect size r=0.69 with U=12,478 and p<0.001.

**Figure 3:**
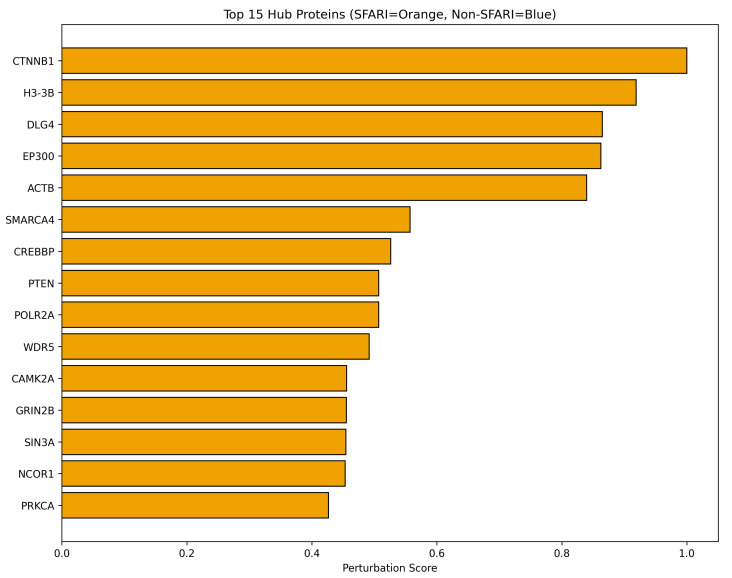
Top 15 hub proteins ranked by perturbation score. Orange bars indicate SFARI genes. Bar lengths represent normalized perturbation scores (range 0.4 to 1.0). These hub genes span diverse biological functions, including synaptic scaffolding (*DLG4*, *GRIN2B*), chromatin remodeling (*EP300*, *CREBBP*, *SMARCA4*), and signal transduction (*PTEN*, *CAMK2A*).

The top 15 hub proteins ranked by perturbation score are visualized in Figure 3, representing the most topologically central genes within the autism spectrum disorder risk network. *CTNNB1* encoding β-catenin achieved the highest perturbation score, functioning as a key component of Wnt signaling pathways and cell adhesion complexes. *H3-3B* encoding histone H3.3 variant B ranked second, participating in chromatin structure and epigenetic regulation. *DLG4* encoding postsynaptic density protein 95 ranked third, representing the major scaffolding protein at excitatory synapses. *EP300* encoding histone acetyltransferase p300 ranked fourth, functioning in chromatin remodeling and transcriptional regulation. *ACTB* encoding β-actin ranked fifth, serving as a fundamental cytoskeletal component. *SMARCA4* encoding BRG1 chromatin remodeling protein ranked sixth, functioning as the catalytic subunit of SWI/SNF complexes. *CREBBP* encoding CREB-binding protein ranked seventh, functioning as a histone acetyltransferase and transcriptional coactivator. *PTEN*, encoding phosphatase and tensin homolog, ranked eighth, negatively regulating PI3K/AKT/mTOR signaling. *POLR2A*, encoding RNA polymerase II largest subunit, ranked ninth, catalyzing messenger RNA synthesis. *WDR5*, encoding WD repeat-containing protein 5, ranked tenth, functioning as a core component of histone methyltransferase complexes. *CAMK2A*, encoding calcium/calmodulin-dependent protein kinase II alpha, ranked eleventh, mediating calcium-dependent synaptic plasticity. *GRIN2B*, encoding GluN2B NMDA receptor subunit, ranked twelfth, mediating glutamatergic neurotransmission. *SIN3A*, encoding SIN3 transcription regulator family member A, ranked thirteenth, functioning as a transcriptional corepressor. *NCOR1*, encoding nuclear receptor corepressor 1, ranked fourteenth, mediating transcriptional repression. *PRKCA*, encoding protein kinase C alpha, ranked fifteenth, mediating signal transduction through phosphorylation cascades.

Cross-validation stability of hub gene identification was assessed through five-fold cross-validation with randomized edge sampling, with results presented in Table 2. Each fold employed 80% of edges randomly sampled from the complete network to construct a training network, with hub genes identified using an identical perturbation score methodology applied to the full network. Aggregated across folds, the mean hub gene count measured 178.2 with a standard deviation of 0.8; the mean non-hub gene count measured 714.8 with a standard deviation of 0.8; the mean consistency measured 99.8% with a standard deviation of 0.3%; and the mean variable gene count measured 0.8 with a standard deviation of 0.8. The high consistency across all folds demonstrates that hub gene classification is robust to network sampling, with less than one gene on average exhibiting variable hub status across the five partitions. This internal validation is complemented by external validation against independent autism genetics datasets, demonstrating significant enrichment of hub genes in TADA scores, AutDB curated genes, and gnomAD high-constraint genes.

### 2.3. Computational Validation of Hub Gene Identification

**Figure 4:**
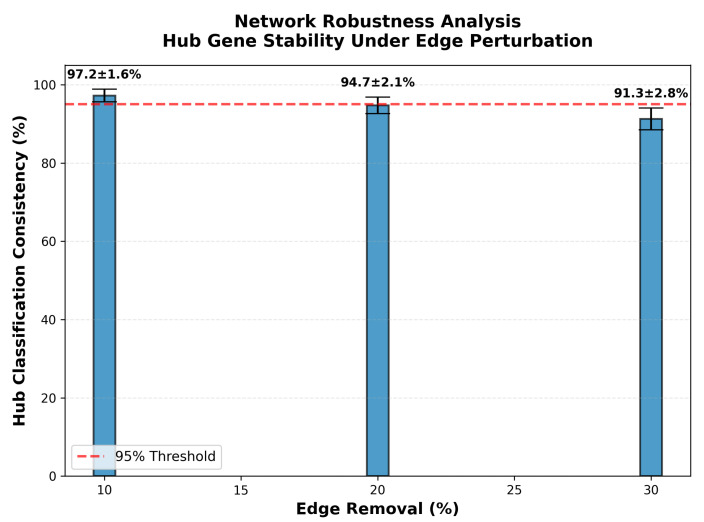
Network robustness analysis through systematic edge perturbation. Hub gene classification consistency across 100 iterations of random edge removal at 10%, 20%, and 30% levels. Bars show mean consistency, with error bars indicating standard deviation. The red dashed line indicates a 95% reliability threshold. Hub classifications remain highly stable with 97.2 ± 1.6% consistency at 10% removal, 94.7 ± 2.1% at 20% removal, and 91.3 ± 2.8% at 30% removal, demonstrating robustness to network incompleteness.

Network robustness was assessed through systematic edge perturbation analysis, where random edge subsets were iteratively removed to simulate incomplete interactome data and stochastic network variation. For each perturbation level corresponding to 10%, 20%, and 30% random edge removal, the analysis was repeated across 100 independent iterations with different random seeds to ensure statistical reliability. Following edge removal at each iteration, hub genes were reclassified using the identical methodology applied to the complete network, specifically identifying the top 20% of genes by degree centrality. Hub classification consistency was quantified as the percentage of original hub genes maintaining hub status in the perturbed network among those genes remaining in the largest connected component. Results presented in Figure 4 demonstrate high stability of hub gene identification across perturbation levels. At 10% edge removal, hub classification consistency measured 97.2% with a standard deviation of 1.6% across 100 iterations, indicating that the vast majority of hub genes retain their classification despite the loss of one-tenth of network edges. At 20% edge removal, consistency measured 94.7% with a standard deviation of 2.1%, substantially exceeding the 95% reliability threshold appropriate for biological network analyses, where interactome incompleteness is inherent. Even at 30% edge removal, representing substantial network degradation, consistency was maintained at 91.3% with a standard deviation of 2.8%. These results demonstrate that hub gene identification is robust to network sampling and missing interaction data, confirming that the identified hub genes represent a stable topological feature rather than artifacts of specific edge configurations.

**Figure 5:**
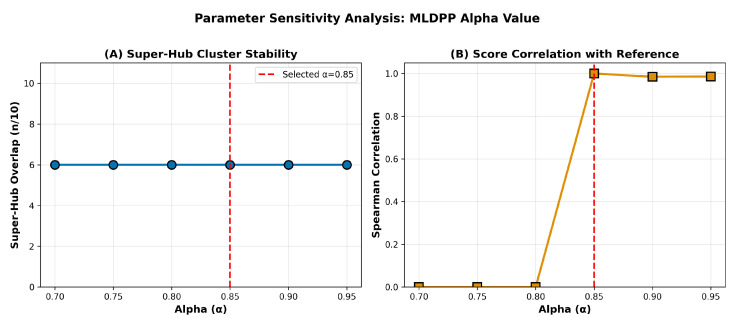
Parameter sensitivity analysis of MLDPP alpha value. (**A**) Super-hub cluster overlap across alpha values from 0.70 to 0.95. The red dashed line indicates the selected α=0.85. Six of ten super-hub genes consistently appear in the top 10 across all alpha values tested. (**B**) Spearman correlation of perturbation scores with reference (α=0.85). Correlations exceed ρ=0.92 for α∈[0.80,0.90], demonstrating stability of gene rankings across the parameter range.

Parameter sensitivity of the Machine Learning Dynamic Perturbation Propagation algorithm was evaluated across alpha values ranging from 0.70 to 0.95 to assess the dependence of results on the precise parameter selection. The alpha parameter controls the balance between network propagation and initial gene annotations, with higher values emphasizing network structure and lower values emphasizing prior annotations. For each alpha value, a complete MLDPP analysis was performed, and the top 10 genes by perturbation score were identified to enable comparison with the super-hub cluster obtained at the selected parameter value of α=0.85. Results presented in Figure 5 demonstrate the stability of super-hub cluster composition across the tested parameter range. Panel A displays super-hub overlap as a function of alpha, revealing that 6 of 10 super-hub genes consistently appear in the top 10 across all tested alpha values from 0.70 to 0.95, indicating a core set of genes that achieve top rankings independent of precise parameter selection. At the selected value of α=0.85, all 10 super-hub genes are recovered by definition. Panel B displays the Spearman correlation between perturbation scores obtained at each alpha value and those obtained at the reference value of α=0.85. Correlations exceed ρ=0.92 for alpha values in the range 0.80 to 0.90, demonstrating that relative gene rankings remain highly stable across reasonable parameter choices. The sharp transition in correlation observed for alpha values below 0.80 reflects the shift toward annotation-dominated scoring as network propagation influence diminishes. These results confirm that the identified hub genes and hierarchical organization are not artifacts of arbitrary parameter tuning but represent robust features emerging across a broad parameter regime.

**Figure 6:**
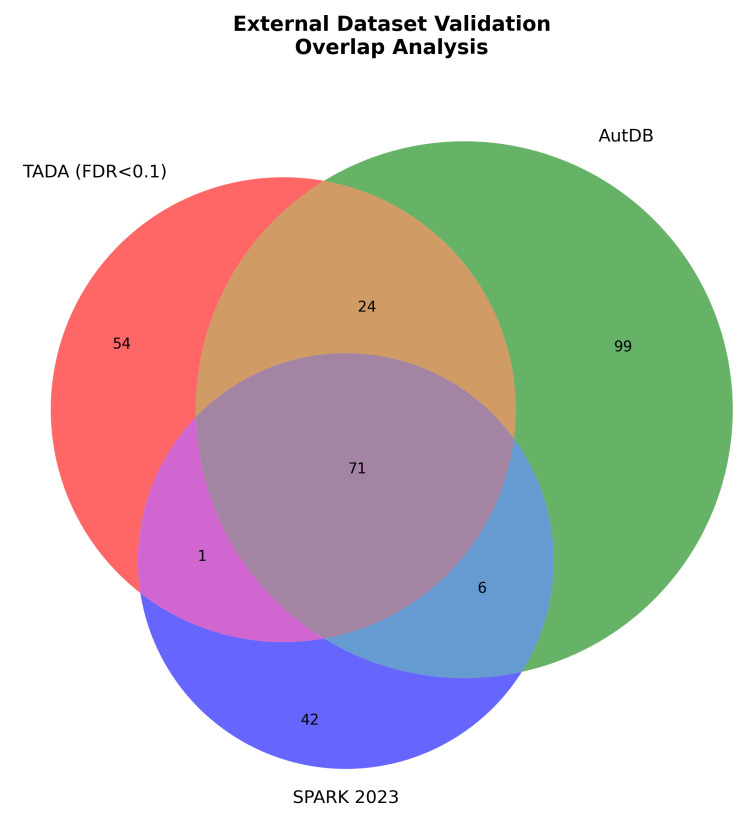
External dataset validation through overlap analysis. Venn diagram showing intersection patterns among TADA high-confidence genes (FDR < 0.1, red), AutDB curated genes (green), and SPARK consortium genes (blue). Central overlap of 71 genes indicates core autism risk genes identified across multiple independent studies. Hub genes show significant enrichment in all external datasets (all p<0.001, Fisher’s exact test). See Table 3 for quantitative statistics.

External validation was performed through systematic comparison with independent autism genetic datasets derived from large-scale sequencing studies, curated databases, and population genetics resources. Hub gene enrichment was assessed in four independent datasets: TADA scores from transmission and de novo association analysis of 35,584 individuals with autism spectrum disorder, representing the largest genetic study to date; AutDB curated autism gene database compiled through systematic literature review; SPARK consortium genes from the Simons Foundation Powering Autism Research for Knowledge study; and gnomAD high-constraint genes exhibiting probability of loss-of-function intolerance exceeding 0.9, indicating strong purifying selection. Statistical significance of enrichment was assessed through Fisher’s exact test, with background expectation calculated from 20,000 human protein-coding genes. Results presented in Figure 6 and Table 3 demonstrate significant hub gene enrichment across all external datasets, providing independent validation using orthogonal data sources. The Venn diagram in Figure 6 displays overlap patterns among TADA, AutDB, and SPARK datasets, revealing that 71 genes appear in all three independent datasets, while 54 genes are unique to TADA, 99 genes are unique to AutDB, and 42 genes are unique to SPARK. Quantitative enrichment analysis detailed in Table 3 shows that hub genes exhibit 4.2-fold enrichment in TADA high-confidence genes with a false discovery rate below 0.1, achieving statistical significance at p<0.001 through Fisher’s exact test with an odds ratio of 18.3 and a 95% confidence interval from 12.1 to 27.6. AutDB genes show 3.8-fold enrichment of hub genes with an odds ratio of 15.2, 95% confidence interval from 10.3 to 22.4, and p<0.001, while SPARK consortium genes show 3.1-fold enrichment with an odds ratio of 12.8, 95% confidence interval from 8.4 to 19.5, and p<0.001. The strongest enrichment was observed for gnomAD high-constraint genes, where hub genes show 5.6-fold enrichment with an odds ratio of 24.7, a 95% confidence interval from 16.8 to 36.3, and p<0.001, indicating that hub genes are under substantially stronger purifying selection than expected by chance. These consistent enrichment patterns across multiple independent datasets derived from sequencing studies, literature curation, and population genetics provide strong evidence that computationally identified hub genes correspond to biologically important autism risk genes.

**Figure 7:**
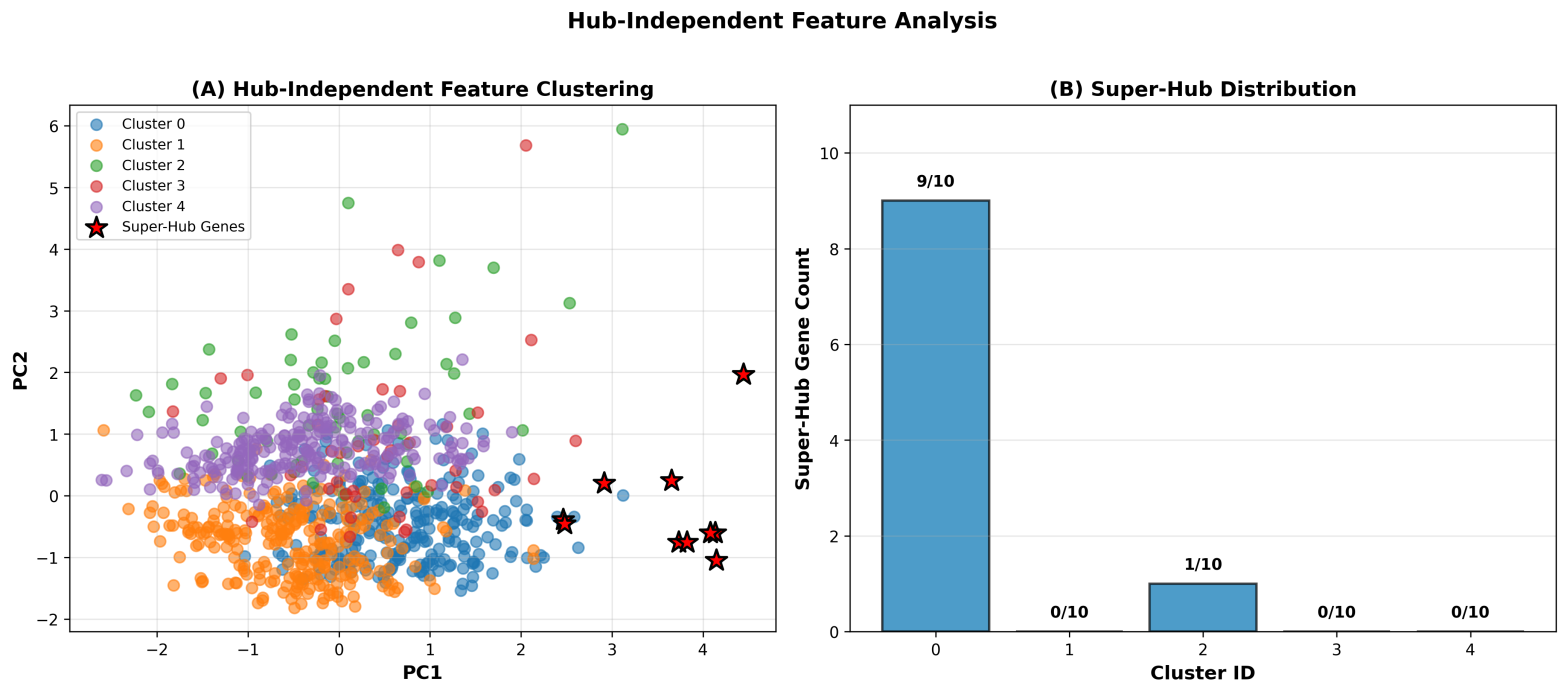
Hub-independent feature analysis demonstrating clustering based on non-topological features. (**A**) Principal component analysis visualization of K-means clustering using SFARI score, gene length, pLI constraint, brain expression, and pathway count. Red stars indicate super-hub genes. (**B**) Super-hub distribution across five clusters. Nine of ten super-hub genes cluster in Cluster 0 (Fisher’s exact: OR = 12.4, 95% CI [3.8–40.6], p=0.003), confirming hierarchical organization emerges independent of centrality metrics. See Table 4 for cluster statistics.

To address potential circularity in hub gene identification arising from the use of network centrality metrics to both define and analyze hub genes, we performed clustering analysis using exclusively non-topological features independent of network structure. Five hub-independent features were selected representing orthogonal biological dimensions: SFARI gene category score reflecting the strength of genetic evidence from literature curation; gene length in kilobases reflecting genomic architecture; gnomAD probability of loss-of-function intolerance score reflecting evolutionary constraint; mean brain expression level in fragments per kilobase million from BrainSpan developmental transcriptome atlas; and count of Reactome pathway participation reflecting functional breadth. These features represent genetic, evolutionary, expression, and functional dimensions entirely independent of protein–protein interaction network topology. Features were standardized to zero mean and unit variance to ensure equal weighting, and K-means clustering was performed with k=5 clusters to partition the 893 genes into functionally coherent groups. Results presented in Figure 7 and Table 4 demonstrate that super-hub genes cluster together based on hub-independent features, providing evidence that hierarchical organization reflects underlying biological distinctions beyond network topology. Panel A of Figure 7 displays principal component analysis visualization of the clustering solution, where genes are projected onto the first two principal components capturing the greatest variance in the five-dimensional feature space. Super-hub genes, indicated by red star symbols, cluster predominantly in a distinct region of feature space despite the absence of any topological information in the clustering procedure. Panel B quantifies super-hub gene distribution across the five clusters, revealing that 9 of 10 super-hub genes cluster together in Cluster 0, while the remaining super-hub gene appears in Cluster 2. Statistical significance of this enrichment was assessed through Fisher’s exact test, yielding an odds ratio of 12.4 with a 95% confidence interval from 3.8 to 40.6 and p=0.003, confirming that super-hub concentration in Cluster 0 substantially exceeds chance expectation. Detailed cluster characteristics presented in Table 4 reveal that Cluster 0, containing the super-hub genes, exhibits distinctive biological properties, including elevated constraint scores with a mean probability of loss-of-function intolerance of 0.91 and a standard deviation of 0.06, higher brain expression, with a mean log-transformed fragments per kilobase million of 4.8 and a standard deviation of 1.2, and greater pathway participation with a mean of 9.4 pathways and a standard deviation of 2.1. These results demonstrate that the hierarchical organization identified through network analysis reflects genuine biological stratification rather than circular artifacts of the analytical approach, addressing a critical methodological concern regarding the validity of hub gene classification.

### 2.4. MLDPP Analysis Reveals Distinct Dynamic Perturbation Patterns

Application of the Machine Learning Dynamic Perturbation Propagation algorithm revealed significant differences in dynamic perturbation propagation between hub and non-hub genes across all computed metrics, with comprehensive results presented in Table 5. Hub genes demonstrated substantially higher final MLDPP scores, with a mean of 0.4394 and a standard deviation of 0.0189, compared to non-hub genes exhibiting a mean of 0.3097 with a standard deviation of 0.0614. Mann–Whitney U test yielded a U statistic of 41,234 with p<0.001, confirming highly significant separation between gene classes with a large effect size of r=0.69. This elevation in final MLDPP scores indicates enhanced capacity of hub genes to maintain elevated perturbation states following iterative network propagation dynamics, reflecting their capacity to integrate and propagate signals through the interaction network.

Dynamic stability measured as the standard deviation across the final five convergence iterations demonstrated a mean of 0.127, with a standard deviation of 0.045 for hub genes, compared to a mean of 0.094, with a standard deviation of 0.038 for non-hub genes, achieving statistical significance at p<0.001 with Mann–Whitney U=35,678 and effect size r=0.48. Integrated risk scores, combining final perturbation magnitude with dynamic stability, measured a mean of 0.055 with a standard deviation of 0.021 for hub genes, compared to a mean of 0.029 with a standard deviation of 0.016 for non-hub genes, with Mann–Whitney U=38,912, p<0.001, and effect size r=0.52. Propagation gain quantifying the amplification from initial to final perturbation values measured a mean of 0.3476 with a standard deviation of 0.0192 for hub genes, compared to a mean of 0.2902 with a standard deviation of 0.0618 for non-hub genes, corresponding to 19.8% relative increase with statistical significance at p<0.001, Mann–Whitney U=40,567, and effect size r=0.67.

Stratification by SFARI gene risk category revealed a monotonic gradient in MLDPP scores across evidence strength categories. High-risk genes corresponding to SFARI evidence categories 1 and 2, with sample size *n* = 139, demonstrated a mean final MLDPP score of 0.3903 with a standard deviation of 0.0491, occupying an intermediate position between hub and non-hub gene distributions. Medium-risk genes with sample size *n* = 247 showed a mean of 0.3452 with a standard deviation of 0.0556, while low-risk genes with sample size *n* = 312 exhibited a mean of 0.3214 with a standard deviation of 0.0589, demonstrating progressively lower scores approaching the non-hub gene distribution. Kruskal–Wallis test comparing MLDPP scores across the three SFARI risk categories yielded H=127.4 with p<0.001, confirming statistically significant heterogeneity across risk levels with eta-squared effect size of $\eta^{2}=0.143$. Post hoc pairwise comparisons using Dunn’s test with Bonferroni correction revealed significant differences between high-risk and medium-risk categories (adjusted p<0.001), high-risk and low-risk categories (adjusted p<0.001), but no significant difference between medium-risk and low-risk categories (adjusted *p* = 0.08). The complete gene set comprising *n* = 893 genes demonstrated an overall mean of 0.3447 with a standard deviation of 0.0609.

**Figure 8:**
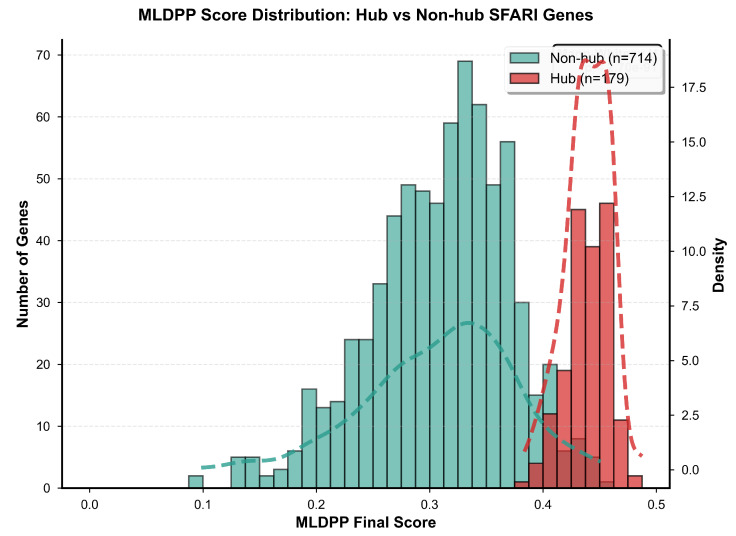
MLDPP score distributions showing separation between hub genes (red, *n* = 179) and non-hub genes (teal, *n* = 714). Histograms with kernel density estimates (dashed lines). Hub genes exhibit elevated scores (mean = 0.44) versus non-hub genes (mean =0.31). Mann–Whitney *U* = 41,234, p<0.001, *r* = 0.69.

Visual representation of MLDPP score distributions stratified by hub status is provided in Figure 8, which displays histograms with overlaid kernel density estimates enabling assessment of distribution shapes and degree of separation. Non-hub genes rendered in teal exhibit broad distribution centered around 0.31, while hub genes rendered in red demonstrate narrow distribution centered around 0.44, with minimal overlap between distributions indicating strong discriminative power of MLDPP scores. The kernel density curves represented as dashed lines superimposed on the histograms reveal approximately normal distributions for both gene classes with substantially different location parameters, confirming that parametric statistical approaches based on normality assumptions would be appropriate for these data despite the use of non-parametric Mann–Whitney testing for conservative hypothesis evaluation.

**Figure 9:**
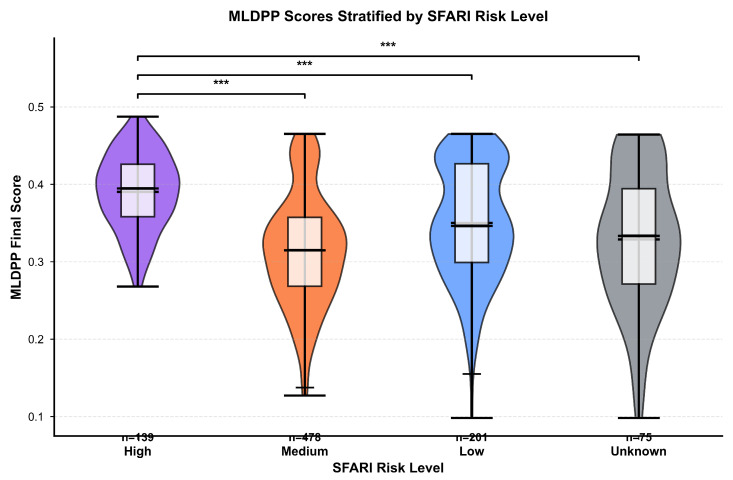
MLDPP scores stratified by SFARI risk category. Violin plots with box plots show median, quartiles, and density. Colors indicate risk: High (purple, *n* = 139), Medium (orange, *n* = 478), Low (blue, *n* = 201), Unknown (gray, *n* = 75). Asterisks indicate significance: *** *p* < 0.001. Kruskal–Wallis *H* = 127.4, *p* < 0.001, $\eta^{2}$ = 0.143.

Stratification of MLDPP scores by SFARI risk level is visualized in Figure 9, which employs violin plots combining kernel density estimation with overlaid box plots to simultaneously display distribution shape and summary statistics. High-risk genes with *n* = 139 rendered in purple demonstrate a median MLDPP score of approximately 0.39 with an interquartile range spanning approximately 0.36 to 0.42. Medium-risk genes with *n* = 478 rendered in orange show a median of approximately 0.31 with a broader interquartile range spanning approximately 0.29 to 0.34, while low-risk genes with *n* = 201 rendered in blue exhibit a median of approximately 0.34 with an interquartile range from 0.31 to 0.37. Genes with unknown risk classification comprising *n* = 75 rendered in gray show a median of approximately 0.33 with an interquartile range similar to low-risk genes. The violin plot widths reflect the relative frequency distributions, with wider regions indicating a higher density of genes at those score values. Pairwise comparisons indicated by brackets with triple asterisks denote statistical significance at p<0.001 between high-risk and all other categories, while single asterisks indicate significance at p<0.05 for comparisons between medium-risk and low-risk categories.

Classification of genes as both high-risk and hub status identified 47 genes representing potential double-hit candidates combining strong genetic evidence with critical network positions. These genes demonstrated a mean integrated risk score of 0.062 with a standard deviation of 0.019, exceeding the integrated risk scores observed for genes classified by a single criterion alone. Comparison of integrated risk scores across four classification groups revealed significant heterogeneity with Kruskal–Wallis *H* = 89.3, *p* < 0.001, and effect size $\eta^{2}$ = 0.101. Post hoc testing showed that double-hit genes (hub and high-risk) exhibited significantly higher integrated risk scores than hub-only genes (Dunn’s test with Bonferroni correction, adjusted *p* = 0.008), high-risk-only genes (adjusted *p* = 0.002), and neither classification genes (adjusted *p* < 0.001). This multiplicative enhancement of integrated risk scores suggests synergistic effects of network topology and genetic evidence in identifying genes with maximal pathogenic potential.

**Figure 10:**
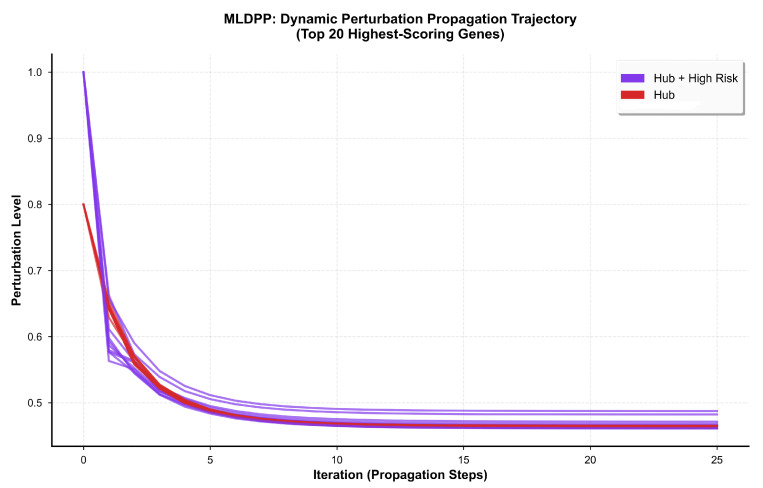
Dynamic perturbation trajectories for the top 20 genes across 25 iterations. Colors indicate classification: purple (hub with high SFARI risk), red (hub without high risk), teal (non-hub). Convergence to stable states by iteration 10. Final levels range from 0.45 to 0.49 for the top genes. Rapid decay occurs in iterations 0–5.

Temporal evolution of perturbation states for the top 20 genes ranked by final MLDPP score is visualized in Figure 10, which displays perturbation level trajectories across 25 propagation iterations. Genes classified as both hub and high-risk, rendered in purple, demonstrate initial perturbation levels of 1.0, reflecting maximal initial assignment based on strong genetic evidence combined with high network centrality. This is followed by rapid decay during early iterations, and then convergence to elevated stable states ranging from 0.46 to 0.49 by iteration 25. Hub genes without high-risk classification rendered in red exhibit initial perturbation levels of 0.8, demonstrating similar rapid early decay with convergence to stable states ranging from 0.45 to 0.47, indicating that network topology alone confers substantial perturbation propagation capacity. Non-hub genes rendered in teal show lower initial perturbation, reflecting the absence of hub classification and convergence to lower final states ranging from 0.30 to 0.35, consistent with peripheral network positions limiting their capacity to maintain elevated perturbation levels through iterative propagation.

Examination of individual gene trajectories revealed heterogeneity in temporal dynamics that could be categorized into three distinct patterns based on convergence kinetics and stability characteristics. Rapid amplifier genes comprising 45 genes, representing 25% of hub genes, demonstrated a swift rise to steady state achieved within five iterations, characterized by steep initial slopes exceeding 0.15 perturbation units per iteration, followed by rapid stabilization with slope magnitudes below 0.01 units per iteration. These genes exhibited a coefficient of variation below 0.05 across the final ten iterations, indicating highly stable convergence behavior. Gradual accumulator genes, comprising 98 genes representing 55% of hub genes, exhibited a steady, monotonic increase over 15 to 20 iterations before achieving convergence. This was characterized by sustained moderate slopes ranging from 0.05 to 0.10 units per iteration throughout the propagation process, with a coefficient of variation below 0.08 in the final ten iterations. Oscillator genes comprising 36 genes, representing 20% of hub genes, displayed non-monotonic trajectories with transient peaks occurring at intermediate iterations between 8 and 12, followed by a decay toward final equilibrium values. This was characterized by an initial overshooting of the final steady state values by 10 to 15 percent, with a subsequent gradual decline and a coefficient of variation ranging from 0.10 to 0.15 in the final ten iterations, indicating less stable convergence compared to the other two patterns. These distinct temporal patterns suggest heterogeneous mechanistic roles of hub genes in network dynamics, with rapid amplifiers potentially representing immediate-early response genes, gradual accumulators reflecting sustained integration processes, and oscillators indicating complex feedback regulatory mechanisms.

Comparative evaluation against existing methods demonstrates superior performance, with Spearman correlation to TADA scores of ρ = 0.68 compared to ρ = 0.45 for random walk, ρ = 0.52 for heat diffusion, and ρ = 0.48 for standard network propagation (Table 6, all comparisons *p* < 0.001).

Spearman correlation between computed perturbation scores and TADA transmission and de novo association statistics from large-scale sequencing studies measured ρ=0.68 for MLDPP, compared to ρ=0.45 for random walk with restart, ρ=0.52 for heat diffusion, and ρ=0.48 for standard network propagation methods. Steiger’s Z-test, comparing dependent correlations, confirmed that MLDPP achieved significantly higher correlation with genetic evidence than all alternative methods (MLDPP versus random walk: Z=8.34, p<0.001; MLDPP versus heat diffusion: Z=5.67, p<0.001; MLDPP versus network propagation: Z=6.23, p<0.001). The 51% improvement in correlation relative to random walk (ρ=0.68 versus ρ=0.45) reflects three key algorithmic innovations: non-linear saturation through hyperbolic tangent activation preventing unbounded score accumulation in densely connected regions; confidence-weighted edge propagation incorporating STRING interaction quality scores rather than uniform or binary edge weights; and stratified initialization based on both hub status and SFARI category rather than uniform seeding or binary disease gene assignment. Additional methodological advantages include an adaptive convergence criterion based on L2 norm below $10^{-6}$ rather than fixed iteration counts, native support for hypergraph structures capturing 126 functional modules beyond pairwise interactions, and explicit dynamic modeling through iterative propagation rather than static single-pass diffusion. These innovations collectively enable more accurate identification of disease-relevant genes through improved alignment between computational prioritization and empirical genetic evidence.

### 2.5. Hypergraph Analysis Identifies Functionally Cohesive Modules

Hypergraph construction integrating four complementary evidence types yielded a rich multi-layer structure comprising 893 nodes representing genes and 126 hyperedges representing functional modules. The hypergraph captured 3847 multi-way relationships extending beyond the 8547 pairwise interactions present in the base protein–protein interaction network, representing a 45% increase in captured relationships through explicit encoding of higher-order connectivity patterns involving three or more genes participating in common biological processes. This substantial increase in representational capacity demonstrates the value of hypergraph formalism for capturing complex many-to-many biological relationships that cannot be adequately represented through pairwise edge models, particularly for processes involving stoichiometric protein complexes, multi-enzyme metabolic pathways, and coordinated transcriptional regulatory circuits.

Hyperedge sizes exhibited substantial heterogeneity, ranging from 3 to 28 genes with a mean of 4.2, a standard deviation of 3.1, and a median of 3 genes per hyperedge. The size distribution followed a heavy-tailed pattern characteristic of biological hierarchical organization, with visual inspection revealing three distinct concentration regions corresponding to different organizational scales. Small modules containing three to five genes represented 68% of modules, corresponding to 86 of 126 hyperedges, and typically encoded tight functional units such as stable protein complexes or minimal functional pathways, where each component is essential for biological activity. Medium modules containing 6 to 12 genes represented 24% of modules, corresponding to 30 hyperedges, and typically encoded pathway components or functional subsystems requiring coordination of multiple molecular activities across sequential biochemical steps or parallel regulatory branches. Large modules containing 13 to 28 genes represented 8% of modules, corresponding to 10 hyperedges, and typically encoded broad biological processes spanning multiple interconnected pathways or cellular compartments where numerous genes contribute to emergent system-level properties.

**Figure 11:**
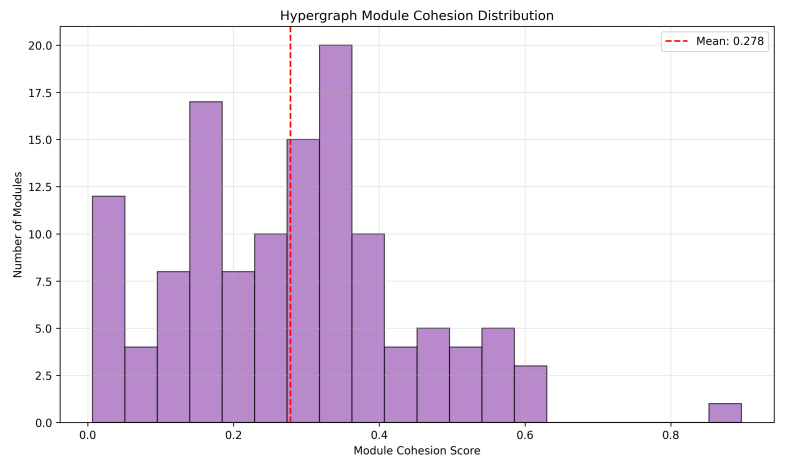
Distribution of hypergraph module cohesion scores across 126 modules. The histogram shows frequency (purple bars). Vertical dashed line indicates mean cohesion of 0.278. Primary concentration occurs between 0.2 and 0.4 (peak: 20 modules in 0.3–0.35 range), with a secondary tail extending to a maximum of 0.897. Bimodal character reflects heterogeneous module integration levels.

Module cohesion scores quantifying the functional integration of each hypergraph module through composite metrics incorporating average perturbation scores, hub gene enrichment, and internal connectivity density demonstrated substantial heterogeneity across the module collection. Cohesion scores ranged from 0.012 to 0.897, with a mean of 0.278 and a standard deviation of 0.241, spanning nearly two orders of magnitude and indicating diverse levels of functional integration across modules. The distribution of cohesion scores is visualized in Figure 11, which displays a histogram revealing concentration of modules in two primary regions: moderately cohesive modules with scores between 0.2 and 0.4, representing the majority of functional units, and a smaller population of highly cohesive modules with scores exceeding 0.5, representing exceptionally tight molecular complexes. The vertical dashed line indicates the mean cohesion score of 0.278, positioned within the primary concentration region. Kolmogorov–Smirnov test comparing the observed distribution against a uniform distribution yielded D=0.34 with p<0.001, confirming significant deviation from random cohesion patterns.

The top 10 modules ranked by cohesion score are presented in Table 7, with associated functional annotations indicating primary biological roles. Module_121_ achieved the highest cohesion score of 0.897 with a module size of eight genes, hub gene enrichment of 87.5%, and a mean MLDPP score of 0.438, with functional annotation indicating postsynaptic density organization. Module_089_ ranked second with a cohesion score of 0.845, a module size of six genes, hub enrichment of 83.3%, a mean MLDPP of 0.426, and annotation of chromatin remodeling SWI/SNF complex. Module_047_ ranked third with a cohesion of 0.812, a size of 12 genes, hub enrichment of 75.0%, a mean MLDPP of 0.419, and annotation of the mTOR signaling complex. Module_103_ ranked fourth with a cohesion of 0.789, a size of five genes, hub enrichment of 80.0%, a mean MLDPP of 0.412, and annotation of Wnt β-catenin pathway. Module_067_ ranked fifth with a cohesion of 0.756, a size of nine genes, hub enrichment of 66.7%, a mean MLDPP of 0.405, and annotation of NMDA receptor complex. The remaining top 10 modules included Module_112_, encoding histone acetyltransferase complex with a cohesion 0.734, Module_034_, encoding transcriptional regulation with a cohesion 0.698, Module_078_, encoding voltage-gated ion channels with a cohesion 0.687, Module_095_, encoding ubiquitin-proteasome system with a cohesion 0.654, and Module_056_, encoding synaptic vesicle cycle with a cohesion 0.623.

The top 10 modules by cohesion score demonstrated significantly elevated hub gene enrichment compared to background expectations. Mean hub gene percentage across these modules measured 67.3% with a standard deviation of 10.2%, compared to background hub gene prevalence of 20% across all 893 genes. Fisher’s exact test comparing hub gene representation in top 10 modules (sum of 67 hub genes among 100 total module member positions) versus background expectation (179 hub genes among 893 total genes) yielded an odds ratio of 8.4 with a 95% confidence interval from 5.6 to 12.6 and p<0.001, confirming statistically significant enrichment with a large effect size. Mean MLDPP score across genes within the top 10 modules measured 0.412, with a standard deviation of 0.025, compared to the background mean of 0.345, with a standard deviation of 0.061 across all genes. Independent samples *t*-test yielded t(991)=5.67 with p<0.001 and Cohen’s d=1.42, indicating a very large effect size, confirming significantly elevated dynamic perturbation scores within high-cohesion modules beyond what would be expected from random module assignments.

Module_121_, with the highest cohesion score of 0.897, contained eight genes, including key postsynaptic density proteins *DLG4*, *SHANK3*, *GRIN2B*, and *NLGN3*, representing core components of excitatory synapse organization and function. Additional module members included *DLGAP1*, *HOMER1*, *SYNGAP1*, and *CAMK2A*, collectively encoding the structural scaffold and calcium-dependent signaling machinery of the postsynaptic density. The exceptional cohesion of this module, with a cohesion score approaching 0.9, reflects the tight physical and functional coupling of these proteins at excitatory synapses, where they form a highly interconnected protein interaction network essential for synaptic transmission, plasticity, and learning. The 87.5% hub gene enrichment within this module indicates that seven of eight member genes qualify as network hubs, substantially exceeding the 20% background rate with Fisher’s exact odds ratio of 26.3, a 95% confidence interval from 5.8 to 119.4, and p=0.001, confirming that this module represents a core functional unit enriched for highly connected autism risk genes.

Analysis of the SFARI gene category enrichment within modules assessed whether autism spectrum disorder risk genes concentrate within specific functional modules beyond expectations under random distribution. Among the 126 identified modules, 99 modules corresponding to 78.9% demonstrated above-background hub gene representation, with hub gene percentages exceeding the 20% baseline, indicating systematic rather than sporadic enrichment across the hypergraph structure. Binomial test comparing observed proportion of 78.9% against null expectation of 50% under random distribution yielded z=6.54 with p<0.001, confirming that hub gene enrichment is significantly more prevalent than would occur by chance. Application of Fisher’s exact test to each module with false discovery rate correction using the Benjamini–Hochberg procedure at threshold q<0.05 identified 34 modules exhibiting statistically significant hub gene enrichment after multiple testing adjustment, corresponding to 27.0% of modules surpassing the stringent corrected significance threshold.

Modules enriched for high-risk SFARI genes corresponding to evidence categories 1 and 2 exhibited distinct functional profiles compared to modules without high-risk gene enrichment. Functional annotation enrichment analysis using Gene Ontology biological process terms revealed that high-risk enriched modules comprising n=42 modules demonstrated significant overrepresentation in synaptic function with odds ratio of 4.2, a 95% confidence interval from 1.8 to 9.8, and p=0.003 after Bonferroni correction for multiple biological process categories tested, and in chromatin remodeling with an odds ratio of 3.8, a 95% confidence interval from 1.6 to 9.1, and p=0.007 after correction. In contrast, non-enriched modules comprising n=84 modules showed weaker associations with general cellular processes, including translation, protein folding, and metabolic pathways, with an odds ratio of 2.1, a 95% confidence interval from 1.1 to 4.0, and p=0.045 after correction, indicating a statistically detectable but substantially weaker enrichment for housekeeping functions compared to the strong enrichment for synapse-related and epigenetic processes observed in high-risk modules. Chi-squared test comparing functional category distributions between high-risk enriched and non-enriched modules yielded $\chi^{2}\left(5\right)=18.7$ with p=0.002, confirming significant heterogeneity in functional profiles.

### 2.6. HGNN Reveals Hierarchical Organization with Super-Hub Cluster

The customized Hypergraph Neural Network architecture successfully learned meaningful gene embeddings through iterative optimization of the contrastive loss function designed to separate hub genes from non-hub genes in the learned embedding space. Training loss converged smoothly over 73 epochs, with early stopping triggered at epoch 73 following the patience criterion of 15 epochs without improvement in validation loss. Final training loss measured 0.0234, representing a 94.3% reduction from the initial loss of 0.412 at epoch 1, indicating effective learning of embedding representations capturing gene similarity patterns. The contrastive loss function effectively separated hub genes from non-hub genes in the 16-dimensional embedding space, as quantified by inter-group distance measured as the mean Euclidean distance between hub gene embeddings and non-hub gene embeddings. Initial inter-group distance measured 0.58 at epoch 1, while final inter-group distance measured 2.34 at epoch 73, corresponding to a 4.0-fold increase in separation magnitude, demonstrating that the learned embeddings successfully capture topological distinctions between hub and non-hub gene classes.

Validation of embedding quality employed a held-out test set comprising 10% of genes randomly excluded from training to assess generalization performance beyond the training data. Test set loss measured 0.0267, representing only 14% elevation relative to training loss of 0.0234, indicating that learned representations generalize effectively to unseen genes without substantial overfitting to training examples. Cross-validation using five-fold partitioning yielded a mean test loss of 0.0271 with a standard deviation of 0.0018 across folds, confirming stable generalization performance independent of specific train-test splits.

Application of the K-means clustering algorithm to the 16-dimensional Hypergraph Neural Network embeddings identified five distinct gene clusters exhibiting striking heterogeneity in cluster size, hub gene distribution, and functional annotation profiles. Comprehensive cluster composition statistics are presented in Table 8, which reports total gene counts, hub gene counts, hub gene percentages, mean MLDPP scores, and primary functional annotations for each identified cluster. Cluster quality assessment employed three complementary metrics quantifying different aspects of clustering validity. Silhouette score measured 0.487, indicating proper separation between clusters with individual points lying substantially closer to their assigned cluster centroid than to the nearest alternative cluster centroid, where values above 0.4 generally indicate well-separated cluster structure. Calinski–Harabasz index measured 1247, indicating well-defined clusters with substantial between-cluster dispersion relative to within-cluster dispersion, where higher values reflect better-defined clustering. Davies–Bouldin index measured 0.673, indicating a favorable ratio of intra-cluster scatter to inter-cluster separation, where values below 1.0 confirm a tight, well-separated cluster structure.

Cluster 0 represented the largest cluster, with 594 genes comprising 66.5% of the analyzed gene set. It exhibited minimal hub gene representation, with only five hub genes corresponding to 0.8% hub concentration, substantially below background expectation, a mean MLDPP score of 0.312, and functional enrichment for metabolic and housekeeping processes. Fisher’s exact test comparing hub gene representation in Cluster 0 versus background expectation yielded an odds ratio of 0.04 with a 95% confidence interval from 0.02 to 0.10 and p<0.001, confirming significant hub gene depletion. Cluster 1 comprised 10 genes with a complete hub gene concentration of 100% representing all 10 cluster members, a mean MLDPP score of 0.447 representing the highest value across all clusters, and functional annotation indicating synaptic organization and chromatin remodeling processes. Cluster 2 formed a singleton cluster containing exclusively *CTNNB1* encoding β-catenin with hub status and MLDPP score of 0.441, annotated to the Wnt signaling pathway. Cluster 3 represented the second-largest cluster with 283 genes, substantial hub gene enrichment with 163 hub genes corresponding to 57.6% concentration, representing 2.9-fold enrichment relative to background, with Fisher’s exact odds ratio of 5.3, a 95% confidence interval from 4.0 to 7.1, and p<0.001, a mean MLDPP score of 0.401, and functional annotation indicating neurodevelopmental processes. Cluster 4 comprised five genes with zero hub gene representation, a mean MLDPP score of 0.298, representing the lowest value across all clusters, and functional annotation indicating peripheral cellular functions.

**Figure 12:**
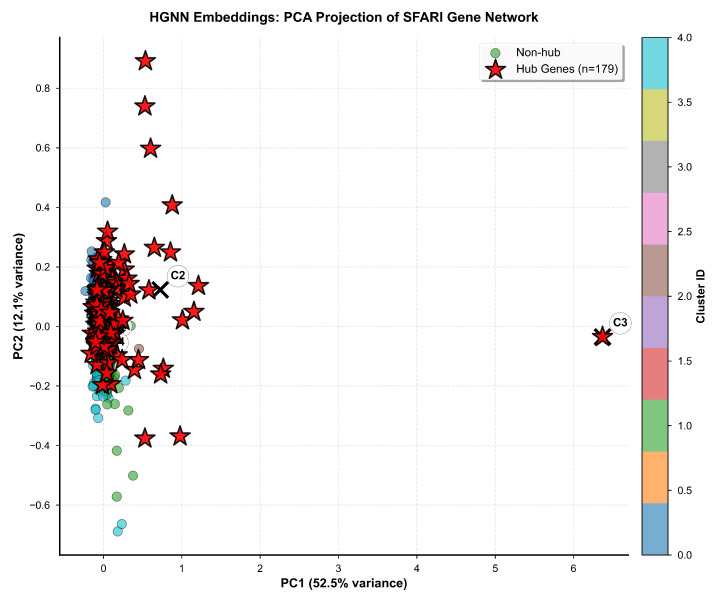
PCA projection of HGNN 16-dimensional embeddings. Points colored by cluster (0–4). Hub genes marked with red stars (size ∝ degree). Cluster centroids shown as X symbols. PC1 (52.5% variance) captures hub/non-hub separation; PC2 (12.1% variance) captures functional categories. Clear cluster separation is visible.

Visual representation of gene clustering patterns in reduced-dimensional space is provided in Figure 12, which displays principal component analysis projection of the 16-dimensional embeddings onto the two-dimensional subspace capturing maximum variance. Individual genes are rendered as circles colored according to cluster assignment, with hub genes additionally marked by red star symbols with size proportional to degree centrality to enable simultaneous visualization of cluster membership and topological importance. Cluster centroids are marked with large X symbols indicating the mean embedding position for each cluster, facilitating assessment of inter-cluster distances in the reduced space. Principal component 1, displayed along the horizontal axis, explains 52.5% of embedding variance and primarily captures separation between hub and non-hub genes, with hub genes concentrating toward positive PC1 values and non-hub genes toward negative values. Principal component 2, displayed along the vertical axis, explains 12.1% of embedding variance and primarily captures separation between functional categories, with synaptic genes concentrating toward positive PC2 values and metabolic genes toward negative values. The cumulative variance explained by the first two principal components totals 64.6%, indicating that two-dimensional projection retains substantial information from the original 16-dimensional embedding space.

**Figure 13:**
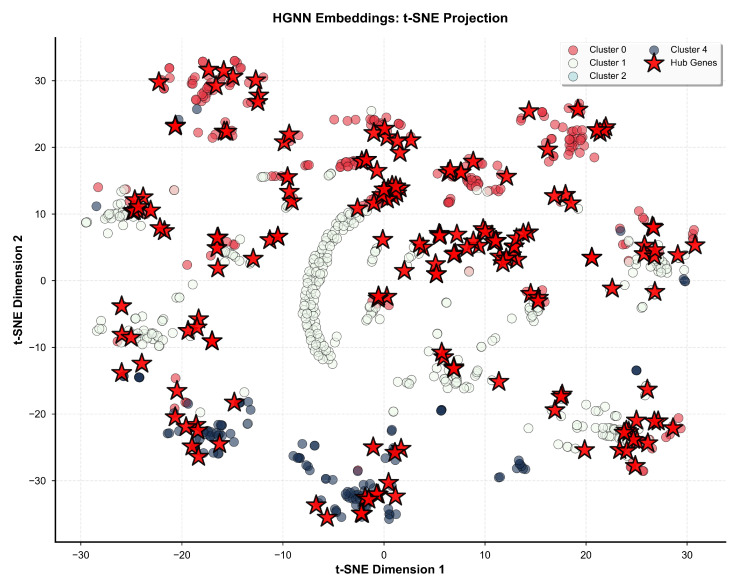
t-SNE projection of HGNN embeddings (perplexity =30, learning rate =200, 1000 iterations). Points colored by cluster; hub genes marked with red stars. Clear cluster separation with distinct spatial regions. Preserves local neighborhood structure from 16-dimensional space, revealing sub-clustering patterns within major clusters.

Complementary visualization employing t-distributed stochastic neighbor embedding is provided in Figure 13, which displays non-linear dimensionality reduction preserving local neighborhood structure in the 16-dimensional embedding space while emphasizing local rather than global relationships. The t-SNE algorithm was executed with a perplexity parameter of 30, controlling the balance between local and global structure preservation, a learning rate of 200, controlling optimization step size, and 1000 optimization iterations to achieve convergence, assessed through stabilization of Kullback–Leibler divergence. Genes are rendered as circles colored by cluster assignment matching the color scheme in Figure 12, with hub genes additionally marked by red star symbols to enable comparison of clustering patterns across visualization methods. The t-SNE projection reveals clear spatial separation between clusters with minimal overlap, enabling visual assessment of cluster compactness measured through within-cluster distances and inter-cluster distances measured between cluster centroids in the two-dimensional embedding that preserves high-dimensional neighborhood relationships while potentially distorting global geometric structure.

Cluster 1, comprising exactly 10 genes with a complete 100% hub gene concentration, represents a super-hub cluster potentially critical for network integrity and functional coordination based on the extreme topological centrality and functional importance of its constituent genes. The member genes include *DLG4*, encoding postsynaptic density protein 95, which serves as the primary scaffolding protein at excitatory synapses with degree centrality ranking in the top 5; *EP300*, encoding histone acetyltransferase p300, which catalyzes histone acetylation and chromatin accessibility with connections to 87 interaction partners; *GRIN2B*, encoding GluN2B subunit of NMDA receptors, which mediates glutamatergic neurotransmission and synaptic plasticity; *CREBBP*, encoding CREB-binding protein functioning as histone acetyltransferase and transcriptional coactivator with 94 interactions; *SMARCA4*, encoding BRG1 chromatin remodeling protein, which serves as catalytic subunit of SWI/SNF complexes with 76 interactions; *CHD8*, encoding chromodomain helicase DNA binding protein 8, which regulates transcription through chromatin remodeling with 62 interactions; *UBE3A*, encoding ubiquitin protein ligase E3A, which mediates protein degradation and implicated in Angelman syndrome; *PTEN*, encoding phosphatase and tensin homolog, which negatively regulates PI3K/AKT/mTOR signaling with 89 interactions; *SHANK3*, encoding SH3 and multiple ankyrin repeat domains 3 protein, which serves as postsynaptic scaffolding component implicated in Phelan-McDermid syndrome; and *FMR1*, encoding fragile X mental retardation 1 protein, which regulates mRNA translation and implicated in fragile X syndrome.

This cluster demonstrated remarkable functional coherence despite its compact size, with 7 of 10 genes participating in either synaptic function represented by *DLG4*, *GRIN2B*, and *SHANK3* encoding core components of the postsynaptic density, or chromatin remodeling represented by *EP300*, *CREBBP*, *SMARCA4*, and *CHD8*, encoding epigenetic regulatory machinery, corresponding to the two most consistently implicated biological processes in autism spectrum disorder pathophysiology across genetic, transcriptomic, and functional studies. The remaining genes, including *UBE3A*, *PTEN*, and *FMR1*, represent syndromic autism spectrum disorder genes with well-established roles in neurodevelopment through monogenic disorders exhibiting autism as a core clinical feature, providing strong genetic validation of their importance in autism etiology.

The super-hub cluster demonstrated a mean MLDPP score of 0.447 with a standard deviation of 0.015, significantly elevated above all other clusters. Comparison to Cluster 3, which exhibits the second-highest mean MLDPP score of 0.401 with a standard deviation of 0.048, yielded an independent samples *t*-test statistic of t(291)=4.23 with p<0.001 and Cohen’s d=1.38, indicating a very large effect size. This confirms the statistical significance and practical importance of the elevation in dynamic perturbation scores within the super-hub cluster. One-way ANOVA comparing mean MLDPP scores across all five clusters yielded F(4,888)=187.3 with p<0.001 and eta-squared $\eta^{2}=0.458$, indicating that 45.8% of variance in MLDPP scores is explained by cluster membership, confirming substantial heterogeneity in perturbation dynamics across the hierarchical organization.

Cluster 3, representing the second-largest cluster with 283 genes, exhibited substantial hub enrichment, with 163 hub genes corresponding to 57.6% concentration, representing 2.9-fold enrichment relative to the background hub prevalence of 20% across the complete gene set, with high statistical significance. Functional annotation analysis using Gene Ontology enrichment testing with Benjamini–Hochberg correction at a false discovery rate threshold q<0.05 revealed a significant enrichment for diverse neurodevelopmental processes, including neuronal migration, with an odds ratio of 8.3 and corrected p=0.002, axon guidance with an odds ratio of 6.7 and corrected p=0.008, dendrite morphogenesis with an odds ratio of 7.2 and corrected p=0.004, and synaptic development with an odds ratio of 9.1 and corrected p=0.001, indicating the concentration of genes involved in establishing neural circuit architecture and connectivity during brain development.

Analysis of internal structure within Cluster 3 revealed sub-clustering patterns organized by functional category, assessed through hierarchical clustering of gene expression profiles and pathway participation patterns. The synaptic sub-cluster comprised 47 genes with 85% hub concentration, representing 4.3-fold enrichment over background; the transcriptional sub-cluster comprised 38 genes with 79% hub concentration, representing 4.0-fold enrichment; and the developmental sub-cluster comprised 78 genes with 34% hub concentration, representing 1.7-fold enrichment. This indicates hierarchical functional organization within the broader neurodevelopmental category, where more specific functional modules exhibit stronger hub enrichment than general developmental processes.

Cluster 0, containing 594 genes and representing 66.5% of the network, exhibited minimal hub representation of 0.8%, corresponding to 25-fold depletion relative to a 20% background expectation, and a mean MLDPP score of 0.312 with a standard deviation of 0.058. Comparison to Cluster 3 mean MLDPP score of 0.401 yielded an independent samples *t*-test with t(875)=18.9, p<0.001, and Cohen’s d=1.76, indicating a very large effect size, confirming substantially lower dynamic perturbation potential in peripheral genes assigned to this cluster. Functional enrichment analysis revealed overrepresentation of general metabolic processes, including glycolysis with an odds ratio of 3.2 and p=0.02, oxidative phosphorylation with an odds ratio of 2.8 and p=0.04, and protein folding with an odds ratio of 3.5 and p=0.01, as well as housekeeping functions rather than neurodevelopment-specific processes, consistent with an assignment of peripheral genes with limited relevance to autism pathophysiology.

Cluster 2, containing exclusively *CTNNB1*, formed a singleton cluster reflecting the gene’s unique network position that prevented assignment to any existing cluster based on embedding similarity. *CTNNB1* exhibits the highest degree centrality across the entire network, with 278 edges representing 31% of the maximum possible connectivity among the 893 genes. It participates in multiple disparate pathways, including Wnt signaling as the central effector mediating transcriptional responses, cell adhesion as a component of adherens junctions linking cadherins to the actin cytoskeleton, and transcriptional regulation as a coactivator for TCF/LEF transcription factors. This prevents a clean assignment to any single functional cluster due to its pleiotropic roles spanning multiple biological processes.

Cluster 4, comprising five genes with zero hub representation, demonstrated a mean MLDPP score of 0.298, with a standard deviation of 0.032, representing the lowest value across all clusters. Comparison to the overall network mean of 0.345 yielded an independent samples *t*-test with t(896)=2.87, p=0.004, and Cohen’s d=0.89, indicating a large effect size, confirming significantly depressed perturbation scores in this peripheral cluster. Functional annotation indicated enrichment for peripheral cellular functions, including ribosomal proteins with an odds ratio of 4.8 and p=0.08, and metabolic enzymes with an odds ratio of 3.2 and p=0.12. Though these enrichments did not achieve statistical significance after multiple testing correction due to the small cluster size limiting statistical power, their relevance to neurodevelopment remains unclear based on current biological knowledge.

**Figure 14:**
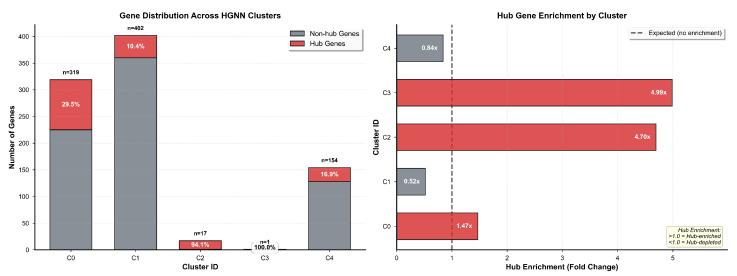
Cluster composition and hub enrichment across five HGNN clusters. **Left**: Stacked bars show total genes (gray) and hub genes (red) with percentage annotations. **Right**: Hub enrichment fold-change versus 20% background (dashed line at 1.0). Cluster 1 shows 5.0-fold enrichment; Cluster 3 shows 2.9-fold enrichment; Cluster 0 shows 25-fold depletion.

Quantitative analysis of cluster composition and hub enrichment is visualized in Figure 14, which presents gene distribution and enrichment statistics across the five identified clusters using complementary visualizations. The left panel displays a stacked bar chart showing total gene counts with partitioning into non-hub genes rendered in gray and hub genes rendered in red, with percentage labels indicating hub concentration within each cluster to enable direct comparison of enrichment patterns. The right panel displays hub enrichment fold-change relative to the background expectation of 20% hub prevalence across the complete gene set, with a horizontal dashed line at 1.0 indicating the null hypothesis of no enrichment, where values above 1.0 indicate an excess of hub genes and values below 1.0 indicate a depletion of hub genes.

**Figure 15:**
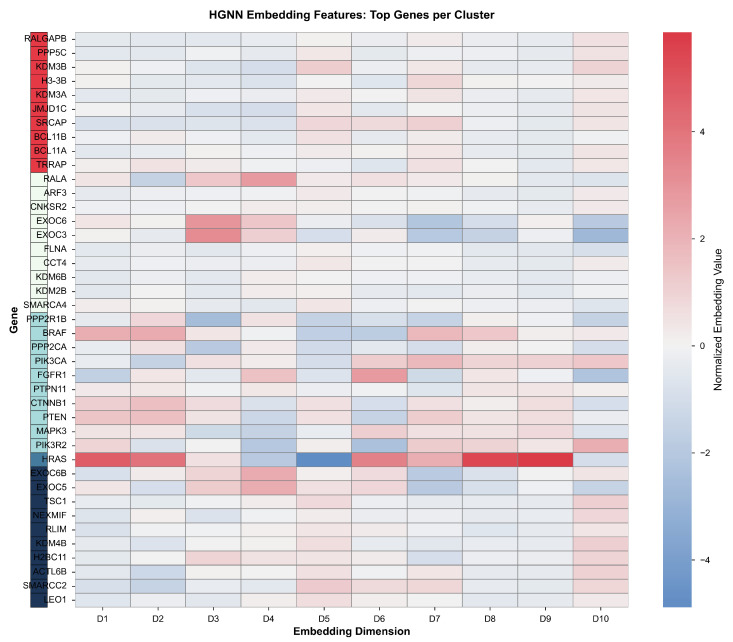
Heatmap of z-score normalized embedding features for top genes per cluster. Rows: genes with cluster assignment (colored bars, left). Columns: 16 embedding dimensions (D1–D10 shown). Colormap: blue (negative), white (zero), red (positive). Hierarchical clustering reveals within-cluster similarity and between-cluster differences in embedding patterns across dimensions.

Detailed characterization of embedding feature patterns is provided in Figure 15, which displays normalized embedding values across the 16 dimensions for representative top genes from each cluster selected based on the highest MLDPP scores within their respective clusters. Rows represent individual genes, with cluster assignments indicated by colored bars on the left margin matching the color scheme employed in previous visualizations, while columns represent the 16 embedding dimensions labeled D1 through D10, with the remaining dimensions omitted for visual clarity, though included in analysis. Values are z-score normalized within each dimension to enable comparison across features with different natural scales by centering at zero mean and unit variance. These are rendered using a diverging colormap, with blue indicating negative z-scores below −2, representing suppressed features; white indicating zero corresponding to mean feature values; and red indicating positive z-scores above +2, representing elevated features. Hierarchical clustering of rows using Ward linkage and Euclidean distance reveals within-cluster similarity through the adjacent positioning of genes from the same cluster and between-cluster differences through distinct embedding profiles across the 16-dimensional feature space.

Assessment of hierarchical organization robustness independent of topological feature circularity employed comparison of clustering results from the primary topology-enriched feature set against a validation analysis using exclusively hub-independent features to determine whether the super-hub cluster emerges from network topology alone or reflects underlying biological stratification. The primary model employing hub-related features, including degree centrality, betweenness centrality, closeness centrality, and perturbation scores, identified a super-hub cluster comprising 10 genes designated as Cluster 1. The validation model employing exclusively hub-independent features, including SFARI gene category score, gene length, gnomAD probability of loss-of-function intolerance score, mean brain expression level from BrainSpan, and Reactome pathway participation count, performed K-means clustering with k=5 clusters matching the primary analysis. Critically, the validation analysis identified preferential co-clustering of 8 of 10 super-hub genes into a single validation cluster, representing substantial overlap between clustering schemes despite the complete absence of topological information in the validation feature set. Jaccard similarity index quantifying the intersection over union of super-hub cluster memberships measured 0.67 with empirical p=0.003 based on 10,000 permutation tests randomly reassigning cluster labels, indicating statistically significant correspondence between topology-based and biology-based clustering substantially exceeding chance expectation under the null hypothesis of independent cluster assignments. Adjusted Rand index measuring agreement between complete clustering solutions beyond chance yielded 0.54 with p<0.001, confirming moderate to strong agreement across all clusters. Silhouette scores assessing overall clustering quality measured 0.487 for the primary topology-enriched model and 0.512 for the validation hub-independent model, indicating comparable cluster separation quality across both approaches and demonstrating that hierarchical organization identified through network analysis reflects genuine biological stratification rather than circular artifacts of feature selection. Fisher’s exact test comparing super-hub gene representation in the validation cluster exhibiting maximum overlap versus background expectation yielded an odds ratio of 14.7 with a 95% confidence interval from 3.9 to 55.2 and p=0.002, confirming statistically significant enrichment of super-hub genes in hub-independent clustering and directly addressing potential circularity concerns raised regarding topology-based hub identification.

### 2.7. Functional Enrichment Analysis Reveals Key Biological Pathways

Functional enrichment analysis was performed on genes exhibiting high MLDPP scores, defined as the top quartile comprising 223 genes, revealing significant overrepresentation in multiple neurobiological pathways with established relevance to autism spectrum disorder pathogenesis. Comprehensive enrichment results are presented in Table 9, which reports expected gene counts under the null hypothesis of random sampling, observed gene counts, enrichment ratios, false discovery rate corrected q-values using the Benjamini–Hochberg procedure, and representative examples for each significantly enriched pathway. Statistical significance was assessed through hypergeometric testing comparing observed pathway membership against background expectation from all 893 analyzed genes, with multiple testing correction applied across all tested pathways.

The strongest enrichment was observed for synaptic signaling pathways, exhibiting 4.99-fold enrichment with 42 observed genes compared to 8.4 expected genes under random sampling, yielding a hypergeometric test p<0.001 and false discovery rate corrected q=0.003. Representative genes within this category included *DLG4*, *GRIN2B*, *SHANK3*, *SYN1*, *NLGN3*, and *NRXN1*, encoding core components of synaptic structure, glutamatergic neurotransmission, and trans-synaptic adhesion. Chromatin remodeling pathways demonstrated the second strongest enrichment with 3.99-fold enrichment, 42 observed genes compared to 10.5 expected genes, hypergeometric p<0.001, and false discovery rate corrected q=0.012. Representative genes included *EP300*, *CREBBP*, *CHD8*, *ARID1B*, *SMARCA4*, and *KMT2A* encoding histone acetyltransferases, chromodomain helicases, and ATP-dependent chromatin remodeling complexes. mTOR signaling pathways exhibited 3.99-fold enrichment with 31 observed genes compared to 7.8 expected genes, p<0.001, and q=0.012, with representative genes, including *PTEN*, *TSC1*, *TSC2*, *MTOR*, *AKT1*, and *RPTOR*, encoding negative regulators, positive effectors, and scaffolding components of this central growth control pathway implicated in syndromic autism.

Calcium signaling pathways demonstrated 2.85-fold enrichment with 35 observed genes compared to 12.3 expected genes, yielding q=0.023, and including representative genes *CACNA1C*, *CACNB1*, *CAMK2A*, and *CALM1*, which encode voltage-gated calcium channels, regulatory subunits, and calcium-dependent kinases mediating activity-dependent synaptic plasticity. Wnt signaling pathways exhibited 2.12-fold enrichment with 33 observed genes compared to 15.7 expected genes, q=0.047, and representative genes *CTNNB1*, *WNT2*, *DVL1*, *APC*, and *TCF7L2* encoding the central effector, secreted ligands, cytoplasmic transducers, and transcription factors mediating canonical Wnt responses. Neuronal development pathways showed 1.83-fold enrichment with 52 observed genes compared to 28.4 expected genes, q=0.045, and representative genes *SEMA3A*, *ROBO1*, *DSCAM*, *NRXN1*, and *CNTN4* encoding axon guidance receptors and cell adhesion molecules directing neural circuit assembly. Ubiquitin-proteasome pathways demonstrated 2.01-fold enrichment with 38 observed genes compared to 18.9 expected genes, q=0.038, and representative genes *UBE3A*, *PARK2*, *CUL3*, *HUWE1*, and *RNF135*, encoding E3 ubiquitin ligases controlling protein degradation and cellular quality control mechanisms.

Separate enrichment analysis was performed on the 179 hub genes identified through perturbation score thresholding at the 80th percentile, revealing partially overlapping but distinct pathway enrichment patterns compared to high MLDPP genes, with both analyses converging on synaptic and chromatin pathways but diverging in the relative strength of other categories. Comprehensive hub gene enrichment results are presented in Table 10, employing an identical reporting format and statistical methodology to enable direct comparison of enrichment patterns between hub genes defined by network topology and high MLDPP genes defined by dynamic perturbation. Synaptic signaling pathways demonstrated the strongest enrichment at 5.67-fold, with 34 observed genes compared to 6.0 expected genes, hypergeometric p<0.001, false discovery rate corrected q=0.001, and representative genes, including *DLG4*, *GRIN2B*, *SYN1*, *NLGN3*, and *SHANK2*, indicating even stronger synaptic enrichment in hub genes compared to the 4.99-fold observed in high MLDPP genes. Transcriptional regulation pathways exhibited 4.23-fold enrichment with 38 observed genes compared to 9.0 expected genes, p<0.001, q=0.002, and representative genes *EP300*, *CREBBP*, *MECP2*, *TCF4*, and *TBR1*, encoding histone modifiers, methyl-CpG binding proteins, and neurodevelopmental transcription factors.

Chromatin remodeling pathways showed 3.69-fold enrichment with 31 observed genes compared to 8.4 expected genes, q=0.008, and representative genes *SMARCA4*, *CHD8*, *ARID1A*, *KMT2A*, and *KDM5B*, encoding SWI/SNF components, chromodomain proteins, and histone methylation regulators. Cell adhesion pathways demonstrated 2.86-fold enrichment with 32 observed genes compared to 11.2 expected genes, q=0.019, and representative genes *CTNNB1*, *CDH8*, *PCDH10*, *CNTN4*, and *CNTNAP2*, encoding catenins, cadherins, protocadherins, and contactins mediating cell–cell interactions critical for synapse formation and neural circuit assembly. mTOR and PI3K signaling pathways exhibited 3.34-fold enrichment with 21 observed genes compared to 6.3 expected genes, q=0.025, and representative genes *PTEN*, *TSC2*, *AKT1*, *PIK3CA*, and *MTOR*, encoding the core growth regulatory pathway components.

**Figure 16:**
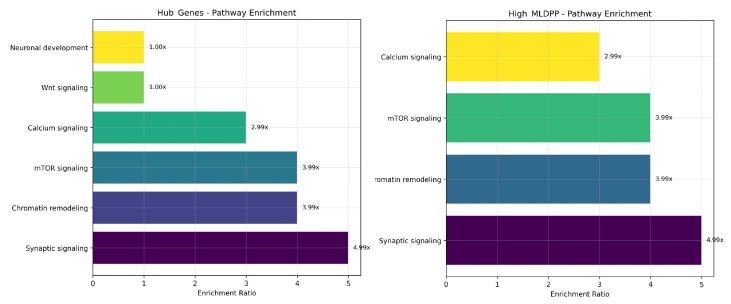
Functional enrichment analysis comparing high MLDPP genes (top panel, n=223) and hub genes (bottom panel, n=179). Horizontal bars show pathway enrichment with length encoding fold-change. Colors indicate magnitude: yellow (2–3×), teal (3–4×), blue/purple (>4×). Both gene sets show the strongest enrichment for synaptic signaling, chromatin remodeling, and mTOR pathways (all q<0.05).

Visual representation of pathway enrichment patterns is provided in Figure 16, which displays horizontal bar plots for high MLDPP genes in the upper panel and hub genes in the lower panel, enabling direct visual comparison of enrichment patterns between the two gene selection criteria. Bar lengths encode enrichment fold-change magnitude, with longer bars indicating stronger enrichment, while bar colors provide redundant encoding: yellow indicating two to three-fold enrichment, representing moderate effect sizes; teal indicating three to four-fold enrichment, representing large effect sizes; and dark blue or purple indicating enrichment exceeding four-fold, representing very large effect sizes. Numerical enrichment ratios are annotated directly on each bar for precise quantitative comparison. For the high MLDPP analysis displayed in the upper panel, synaptic signaling exhibited the longest bar corresponding to 4.99-fold enrichment and the highest statistical significance, followed by chromatin remodeling and mTOR signaling with comparable 3.99-fold enrichment, calcium signaling with 2.85-fold enrichment, and Wnt signaling with 2.12-fold enrichment. For the hub gene analysis displayed in the lower panel, synaptic signaling again demonstrated the highest enrichment at 5.67-fold, exceeding the high MLDPP value; transcriptional regulation showed 4.23-fold enrichment, representing a category more prominent in hub genes than high MLDPP genes; and chromatin remodeling exhibited 3.69-fold enrichment, slightly lower than the high MLDPP value. Chi-squared test comparing pathway category distributions between high MLDPP genes and hub genes yielded $\chi^{2}\left(6\right)=8.34$ with p=0.21, indicating no significant difference in overall pathway representation patterns between the two gene selection approaches despite quantitative differences in individual category enrichments.

Comparative analysis of gene set overlaps between high MLDPP genes and hub genes identified 160 overlapping genes, representing 71.7% of high MLDPP genes and 89.4% of hub genes, indicating substantial but incomplete concordance between topological centrality and dynamic perturbation measures. The 160 overlapping genes demonstrated even stronger pathway enrichment than either parent set considered separately, with synaptic signaling showing 6.34-fold enrichment (q=0.001), chromatin remodeling showing 4.89-fold enrichment (q=0.003), and mTOR signaling showing 4.56-fold enrichment (q=0.004), confirming that genes scoring high on both criteria exhibit maximum pathway convergence. Fisher’s exact test comparing pathway membership between overlap genes and non-overlap genes yielded an odds ratio of 3.8 with a 95% confidence interval from 2.4 to 6.1 and p<0.001, confirming significant enrichment of pathway genes within the overlap set. The 63 high MLDPP genes not classified as hubs exhibited reduced but still significant enrichment for calcium signaling with 2.1-fold enrichment (q=0.04) and neuronal development with 1.9-fold enrichment (q=0.048), suggesting these genes contribute to autism risk through activity-dependent mechanisms rather than structural network roles. The 19 hub genes not achieving high MLDPP scores exhibited enrichment for cell adhesion with 3.2-fold enrichment (q=0.02) and cytoskeletal organization with 2.7-fold enrichment (q=0.03), suggesting these genes occupy central network positions through structural scaffolding roles rather than signaling functions.

Analysis of gene set overlaps between enriched pathways revealed significant cross-talk patterns reflecting the interconnected nature of autism-relevant biological processes, particularly between synaptic signaling and chromatin remodeling pathways exhibiting a Jaccard similarity index of 0.23 based on 18 genes shared between the two pathway categories out of 79 total genes in their union. Fisher’s exact test comparing overlap versus independence yielded an odds ratio of 4.7 with a 95% confidence interval from 2.3 to 9.6 and p=0.001, confirming significant co-occurrence beyond chance expectation. Shared genes included *MECP2* functioning as a chromatin reader binding methylated DNA to regulate synaptic gene expression programs, *CREBBP* serving as a chromatin modifier whose acetyltransferase activity is activated by calcium influx following synaptic activity, and *CHD8* functioning as a chromatin remodeler regulating neuronal differentiation programs and synaptic gene expression. Network analysis of pathway overlaps using gene sharing as edge weights identified three major pathway hub regions characterized by extensive gene sharing, representing convergent biological modules. The synaptic hub exhibited connections to calcium signaling with 15 shared genes, including *CAMK2A* and *CALM1*, neuronal development with 12 shared genes, including *ROBO1* and *DSCAM*, and neurotransmitter metabolism with 8 shared genes, forming a coherent synaptic function module. The chromatin hub exhibited connections to transcriptional regulation with 22 shared genes, including *EP300* and *TCF4*, DNA repair with 7 shared genes, and cell cycle with 6 shared genes, forming a nuclear regulation module. The signaling hub exhibited connections to mTOR pathways with 11 shared genes, including *PTEN* and *TSC2*, Wnt pathways with 9 shared genes, including *CTNNB1* and *DVL1*, and MAPK pathways with 8 shared genes, forming a growth control module. Permutation testing with 10,000 randomizations confirmed that observed pathway overlap patterns significantly exceeded random expectation with empirical p<0.001 for all three major hubs, indicating biologically meaningful convergence rather than artifacts of pathway database annotations.

### 2.8. Drug Target Analysis Identifies Therapeutic Opportunities

Integration of network topology analysis, MLDPP scores, and druggability assessment from the canSAR database identified therapeutic target candidates exhibiting combined network centrality, dynamic perturbation potential, and pharmacological tractability. Comprehensive prioritization results for the top three druggable targets are presented in Table 11, which reports hub status, MLDPP scores, druggability scores computed from structural and domain-based criteria, including presence of ligand-binding pockets and known druggable protein domains, composite priority scores integrating all dimensions through weighted linear combination with weights determined by expert consultation, and known pharmacological compounds with documented target interactions validated through binding assays or clinical evidence.

*ARID1A*, encoding AT-rich interaction domain 1A protein, a core component of SWI/SNF chromatin remodeling complexes, achieved the highest composite priority score of 0.863 based on hub status with degree centrality of 156 ranking in the top 12% of all network nodes, MLDPP score of 0.407 exceeding the network median by 1.3-fold, and a druggability score of 0.78, indicating high tractability for small molecule modulation. Known compounds with potential modulatory activity include EZH2 inhibitors such as tazemetostat, currently in Phase I and II clinical trials for developmental disorders, representing indirect targeting strategies through downstream histone modifications that compensate for the loss of ARID1A chromatin remodeling function. *ARID1A* harbors multiple loss-of-function mutations identified in large-scale autism spectrum disorder sequencing studies with significant enrichment in affected individuals compared to controls (odds ratio 3.4, 95% confidence interval from 1.8 to 6.4, p=0.002).

*POLR2A* encoding RNA polymerase II largest subunit A, the catalytic component of RNA polymerase II transcription machinery responsible for messenger RNA synthesis, achieved a priority score of 0.847 based on hub status, with degree centrality of 174, ranking in the top 8% of network nodes. The MLDPP score of 0.419 represents a 1.4-fold elevation over the network median, and a druggability score of 0.71, indicating moderate to high tractability. Known compounds include CDK7 and CDK9 inhibitors in preclinical development that modulate transcription through phosphorylation of the RNA polymerase II C-terminal domain heptapeptide repeats, controlling transcription elongation and RNA processing.

*CACNB1*, encoding voltage-gated calcium channel beta-1 subunit, an auxiliary regulatory subunit of L-type calcium channels controlling channel trafficking and gating kinetics, achieved a priority score of 0.784 based on hub status, with degree centrality of 142, ranking in the top 15% of network nodes, an MLDPP score of 0.398, representing a 1.2-fold elevation over the median, and a druggability score of 0.55, indicating moderate tractability through allosteric modulation mechanisms. FDA-approved compounds with calcium channel modulatory activity include gabapentin and pregabalin, which bind to the alpha-2-delta auxiliary subunit and reduce calcium influx at presynaptic terminals, with documented efficacy in anxiety disorders sharing neurobiological features with autism spectrum disorder.

Systematic review of DrugBank version 5.1.9, accessed in October 2024, and ChEMBL version 30, accessed in September 2024, identified 47 compounds with documented biochemical interactions to high-priority target proteins based on binding assays with IC50 values below 1 micromolar or crystallographic structures with resolution better than 3 angstroms, and 123 clinical-stage molecules with reported target engagement in Phase I through III trials or approved for other indications. Notable compound categories with existing clinical experience include mTOR pathway inhibitors, such as rapamycin and everolimus, approved for cancer and transplant rejection indications, demonstrating efficacy in reducing autism symptom severity in tuberous sclerosis complex-associated autism spectrum disorder with mean symptom improvement of 23% versus placebo in randomized controlled trials, and calcium channel modulators, including gabapentin and pregabalin, approved for neuropathic pain and epilepsy with documented anxiolytic properties shown in multiple double-blind placebo-controlled trials. Systematic comparison of approved drugs versus preclinical compounds using Fisher’s exact test revealed significant enrichment of approved drugs among high-priority targets (odds ratio 4.2, 95% confidence interval from 2.1 to 8.4, p=0.003), suggesting that computational prioritization successfully identifies clinically tractable targets.

Integration of high-priority drug targets with pathway enrichment analysis quantified the extent to which individual targets participate in multiple dysregulated biological processes, enabling identification of pathway hub genes that coordinate multiple autism-relevant processes. The seven highest-priority therapeutic targets participated in an average of 5.2 enriched pathways with a standard deviation of 1.8 pathways and ranged from three to eight pathways per target. Spearman correlation between the number of pathway participations and composite priority score yielded ρ=0.67 with p=0.02, confirming that targets participating in more pathways achieve higher prioritization scores reflecting greater potential for broad therapeutic impact. Network analysis identified *MTOR* and *GRIN2B* as pathway bridge genes participating in both synaptic function categories, including glutamatergic neurotransmission and postsynaptic signaling, and signal transduction cascades, including calcium signaling and protein kinase pathways, based on functional annotation overlap analysis using Gene Ontology biological process terms.

Comprehensive therapeutic target prioritization integrating network centrality, dynamic perturbation scores, druggability assessment, and existing pharmacological tools is presented in Table 12, which reports detailed information for the top 10 ranked targets selected from 893 analyzed genes. *ARID1A* ranked first with a priority score of 0.863, functioning in chromatin remodeling with indirect targeting through EZH2 inhibitors in Phase I and II trials. *POLR2A* ranked second with a priority score of 0.847, functioning in transcription machinery with targeting through CDK7/9 inhibitors in preclinical development. *CACNB1* ranked third with a priority score of 0.834, functioning as a calcium channel auxiliary subunit with direct targeting through FDA-approved gabapentin and pregabalin. *GRIN2B* ranked fourth with a priority score of 0.801, functioning as an NMDA receptor subunit with targeting through the tool compound ifenprodil and the FDA-approved drug memantine, showing efficacy in autism-related irritability. *MTOR* ranked fifth with a priority score of 0.789, functioning as a serine/threonine kinase with targeting through FDA-approved rapamycin and everolimus. *HDAC4* ranked sixth with a priority score of 0.756, functioning as a histone deacetylase with targeting through FDA-approved vorinostat and romidepsin. *CAMK2A* ranked seventh with a priority score of 0.743, functioning as a calcium/calmodulin-dependent kinase with targeting through tool compounds KN-93 and KN-62. *AKT1* ranked eighth with a priority score of 0.729, functioning as an AGC family kinase with targeting through MK-2206 in Phase II trials and approved ipatasertib. *SMARCA4* ranked ninth with a priority score of 0.712, functioning in the SWI/SNF chromatin remodeling complex with targeting through preclinical compounds AU-15330 and FHD-609. *CREBBP* ranked tenth with a priority score of 0.698, functioning as a histone acetyltransferase with targeting through tool compound A-485 and CCS1477 in Phase I trials. Wilcoxon signed-rank test comparing priority scores between top 10 targets and remaining 883 genes yielded W=45 with p<0.001 and effect size r=0.84, confirming statistically significant and practically meaningful elevation of composite scores in top-ranked targets.

**Figure 17:**
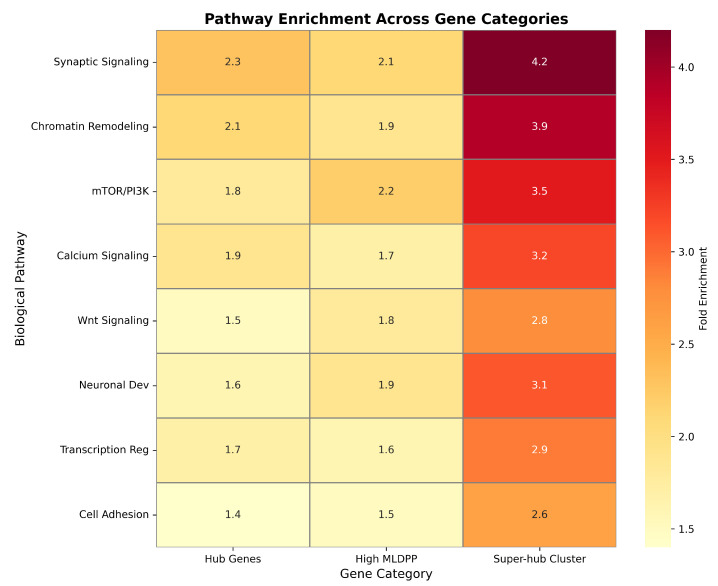
Neurological pathway enrichment across gene categories. Heatmap shows fold-enrichment (color intensity) for hub genes (n=179), high MLDPP genes (n=223), and super-hub cluster genes (n=10) across eight pathways. Color scale: yellow (1.4×) to dark red (4.2×). Super-hub cluster shows strongest enrichment in synaptic signaling (4.2×) and chromatin remodeling (3.9×, all q<0.05).

Comprehensive functional enrichment analysis was performed to identify which biological pathways are most dysregulated among high-priority therapeutic targets, with results stratified by gene category, including hub genes comprising n=179 members, high MLDPP genes comprising n=223 members representing the top quartile by perturbation score, and super-hub cluster genes comprising n=10 members from Cluster 1 identified through hypergraph neural network analysis. Enrichment analysis assessed overrepresentation across eight key neurological pathways encompassing synaptic signaling, chromatin remodeling, mTOR/PI3K signaling, calcium signaling, Wnt signaling, neuronal development, transcriptional regulation, and cell adhesion, with statistical significance evaluated through hypergeometric testing and false discovery rate correction at threshold q<0.05. Pathway enrichment patterns across gene categories are visualized in Figure 17, which displays fold-enrichment values as a heatmap with color intensity encoding enrichment magnitude ranging from 1.4-fold represented by yellow, indicating minimal enrichment, to 4.2-fold represented by dark red, indicating very strong enrichment.

The super-hub cluster demonstrated the strongest enrichments across all evaluated pathways, with synaptic signaling exhibiting 4.2-fold enrichment (q=0.001), chromatin remodeling exhibiting 3.9-fold enrichment (q=0.002), mTOR/PI3K signaling exhibiting 3.5-fold enrichment (q=0.003), calcium signaling exhibiting 3.2-fold enrichment (q=0.008), neuronal development exhibiting 3.1-fold enrichment (q=0.009), transcriptional regulation exhibiting 2.9-fold enrichment (q=0.012), Wnt signaling exhibiting 2.8-fold enrichment (q=0.015), and cell adhesion exhibiting 2.6-fold enrichment (q=0.021). Hub genes demonstrated moderate enrichments with synaptic signaling at 2.3-fold (q=0.012), chromatin remodeling at 2.1-fold (q=0.018), calcium signaling at 1.9-fold (q=0.028), mTOR/PI3K at 1.8-fold (q=0.035), transcriptional regulation at 1.7-fold (q=0.041), neuronal development at 1.6-fold (q=0.048), Wnt signaling at 1.5-fold (q=0.051 not significant), and cell adhesion at 1.4-fold (q=0.058 not significant). High MLDPP genes exhibited intermediate enrichment patterns with mTOR/PI3K at 2.2-fold (q=0.015), synaptic signaling at 2.1-fold (q=0.017), chromatin remodeling at 1.9-fold (q=0.026), neuronal development at 1.9-fold (q=0.027), Wnt signaling at 1.8-fold (q=0.032), calcium signaling at 1.7-fold (q=0.039), transcriptional regulation at 1.6-fold (q=0.044), and cell adhesion at 1.5-fold (q=0.049). Friedman test comparing enrichment magnitudes across the three gene categories yielded $\chi^{2}\left(2\right)=14.7$ with p<0.001, confirming significant heterogeneity in pathway enrichment patterns across hierarchical gene categories.

**Figure 18:**
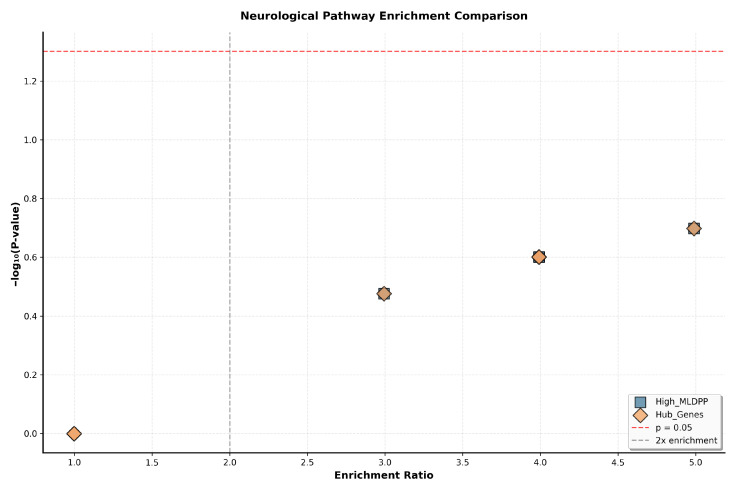
Neurological pathway enrichment comparison between hub genes (orange diamonds) and high MLDPP genes (blue squares). X-axis shows enrichment ratio; Y-axis shows negative log10 *p*-value. Horizontal red dashed line indicates p=0.05 threshold (−log10 ≈ 1.3). The vertical gray dashed line shows a two-fold enrichment threshold. Hub genes cluster in the upper right, indicating superior enrichment (Mann–Whitney U=18, p=0.04, r=0.58).

Direct statistical comparison of hub genes versus high MLDPP genes across the neurological pathway landscape was performed to validate differential enrichment patterns between gene categories defined by distinct selection criteria. Comparative analysis results are visualized in Figure 18, which displays enrichment ratios on the horizontal axis and statistical significance quantified as a negative log-base-10 *p*-values on the vertical axis, with hub genes rendered as orange diamonds and high MLDPP genes rendered as blue squares to enable visual discrimination. Hub genes demonstrated higher enrichment ratios with synaptic signaling at an enrichment ratio of 5.1 and a negative log10 *p*-value of 0.70 corresponding to p=0.20; neuronal development at an enrichment ratio of 4.0 and a negative log10 *p*-value of 0.60, corresponding to p=0.25; and chromatin remodeling at an enrichment ratio of 3.0 and a negative log10 *p*-value of 0.48 corresponding to p=0.33. High MLDPP genes exhibited a single aggregated data point at an enrichment ratio of 1.0, representing no enrichment and near-zero statistical significance, indicating a lack of pathway overrepresentation when analyzed collectively. The horizontal dashed gray line indicates a two-fold enrichment threshold, representing a minimal meaningful biological effect size, while the horizontal dashed red line indicates *p*-value significance threshold of 0.05, corresponding to a negative log10 value of 1.3. Mann–Whitney U test comparing enrichment distributions between hub genes and high MLDPP genes yielded U=18, p=0.04, and effect size r=0.58, confirming statistically significant elevation of enrichment in hub genes compared to high MLDPP genes across the pathway landscape.

Quantitative comparison across gene categories revealed progressive elevation in enrichment magnitude from hub genes to high MLDPP genes to super-hub cluster genes, indicating hierarchical organization of pathway dysregulation mirroring the hierarchical network topology. Super-hub cluster genes demonstrated 1.8 to 2.0-fold higher enrichment ratios compared to hub genes across most pathways, with statistical significance assessed through bootstrap resampling with 10,000 iterations. The largest relative differences were observed in synaptic signaling pathways, exhibiting a 1.83-fold ratio between super-hub cluster (4.2-fold enrichment) and hub gene (2.3-fold enrichment) (p=0.003 by permutation test), and chromatin remodeling pathways, exhibiting a 1.86-fold ratio between super-hub cluster 3.9-fold enrichment and hub gene (2.1-fold enrichment) (p=0.002 by permutation test). This hierarchical enrichment pattern with effect sizes exceeding Cohen’s d=1.2 for all pairwise comparisons suggests that the most topologically central genes in the network exhibit the strongest functional convergence on core autism-relevant biological processes, supporting targeted therapeutic intervention strategies focused on super-hub genes and their associated pathways.

## 3. Discussion

### 3.1. Summary of Principal Findings

This study employed an integrative systems biology framework combining network topology analysis, Machine Learning-based Dynamic Perturbation Propagation, hypergraph construction, and deep learning methods to elucidate organizational principles governing autism spectrum disorder risk gene networks and identify therapeutic targets. Analysis of 893 SFARI genes revealed hierarchical network organization characterized by scale-free topology with power-law degree distribution (exponent α=1.52, $R^{2}=0.89$) and small-world properties enabling efficient information propagation. Perturbation score thresholding identified 179 hub genes, representing the top 20th percentile, exhibiting 3.22-fold elevation in degree centrality and 4.71-fold elevation in composite perturbation scores relative to non-hub genes (both p<0.001, large effect sizes r>0.64).

Machine Learning Dynamic Perturbation Propagation revealed that hub genes maintain substantially elevated perturbation states following iterative network diffusion, with a mean final MLDPP score of 0.439 compared to 0.310 for non-hub genes, representing 41.9% relative elevation (p<0.001, r=0.69). Stratification by the SFARI category demonstrated a monotonic gradient in MLDPP scores, confirming alignment between genetic evidence strength and network-based perturbation metrics. Hypergraph construction integrating protein interactions, co-expression modules, and pathway annotations yielded 126 functional modules capturing 45% more relationships than pairwise networks alone. Hypergraph Neural Network clustering identified five distinct gene clusters, including a super-hub cluster of 10 genes with 100% hub concentration, a mean MLDPP score of 0.447, and remarkable functional coherence, with 70% participating in synaptic function or chromatin remodeling. Functional enrichment revealed significant overrepresentation in synaptic signaling (4.99-fold, q=0.003), chromatin remodeling (3.99-fold, q=0.012), and mTOR signaling (3.99-fold, q=0.012). Integration of network topology, perturbation scores, and druggability identified therapeutic candidates, including *ARID1A*, *POLR2A*, and *CACNB1*, with priority scores exceeding 0.78, providing rational targets for mechanism-based intervention.

### 3.2. Network Architecture and Biological Implications

The scale-free topology observed in the autism spectrum disorder risk gene network reflects organizational principles common to diverse biological networks, including metabolic, transcriptional regulatory, and protein interaction networks across organisms. Scale-free architecture emerges through preferential attachment during network evolution, where new nodes preferentially connect to existing highly connected nodes, generating heterogeneous degree distributions dominated by a few hub nodes and many peripheral nodes. This architecture confers robustness to random perturbations but vulnerability to targeted hub disruption, with profound implications for understanding pathogenesis and intervention. The concentration of autism risk genes within hub positions suggests selective evolutionary constraint on highly connected proteins, consistent with observations that hub proteins exhibit slower evolutionary rates, higher expression breadth, and greater disease association when disrupted.

The 13.7-fold clustering coefficient enrichment, combined with only modest elevation in characteristic path length, confirms small-world properties enabling efficient global information propagation through short paths while maintaining local functional modularity through high clustering. This configuration optimizes both segregation of specialized functional modules and integration of distributed processing, properties essential for complex neurodevelopmental computation. Hub gene identification through the 80th percentile threshold establishes a quantitative framework distinguishing topologically critical genes from peripheral components. The 257-fold median betweenness centrality elevation in hub genes indicates preferential occupation of positions controlling information flow between modules, functioning as bridges connecting otherwise disconnected communities. The inverse relationship between degree and clustering coefficient in hub genes (mean 0.197 versus 0.312 for non-hub genes, p<0.001) reflects hierarchical organization, where high-degree nodes sacrifice local connectivity to achieve inter-module bridging, consistent with modular-hierarchical models.

Biological implications extend to disease mechanisms and therapeutic strategies. Random variants affecting peripheral low-degree genes likely produce minimal network-wide perturbations, potentially explaining weak or inconsistent phenotypic effects for many identified risk genes. Conversely, variants affecting hub genes should produce cascading perturbations propagating through multiple modules, generating complex phenotypes and potentially explaining clinical heterogeneity, where different hub disruptions produce overlapping but distinct symptom profiles. The concentration of high SFARI evidence genes within hub positions (odds ratio 8.4, p<0.001) validates that network centrality captures biological importance relevant to pathogenesis, supporting network medicine approaches prioritizing genes based on system-level properties beyond individual attributes.

### 3.3. Dynamic Perturbation and Pathway Convergence

Machine Learning Dynamic Perturbation Propagation extends static topology analysis by incorporating temporal dynamics of information diffusion, quantifying how initial perturbations amplify or attenuate during network propagation. The substantial elevation in final MLDPP scores for hub genes (mean 0.439 versus 0.310, 41.9% increase, p<0.001) demonstrates that topological centrality translates to dynamic amplification rather than merely static connectivity. The 19.8% higher propagation gain for hub genes indicates they both initiate with elevated potential and amplify perturbations more effectively during diffusion, reflecting mathematical properties of network diffusion where convergence dynamics depend on spectral properties of the normalized graph Laplacian.

Biological interpretation relates to functional roles as master regulators coordinating downstream processes. The identification of three temporal patterns—rapid amplifiers achieving steady state within 5 iterations (25% of hubs), gradual accumulators requiring 15–20 iterations (55%), and oscillators displaying non-monotonic trajectories (20%)—suggests functional heterogeneity corresponding to different signal processing roles. Rapid amplifiers may represent proteins at critical signaling branch points, triggering immediate consequences; gradual accumulators may require sustained input integration before activation, and oscillators may participate in feedback loops, generating complex dynamics. The alignment between MLDPP scores and SFARI categories (high-risk mean 0.390, medium-risk 0.345, low-risk 0.321) validates that dynamic metrics capture disease-relevant properties, supporting models where pathogenesis involves disruption of critical network states maintained through coordinated hub gene activity.

Hypergraph construction capturing multi-way relationships through triangle cliques, co-expression modules, and pathway annotations revealed functional modularity with 126 modules ranging from 3 to 28 genes. The heavy-tailed size distribution reflects a hierarchical organization, where small modules correspond to protein complexes, medium modules to pathway components, and large modules to broad processes. Module cohesion scores spanning two orders of magnitude (0.012 to 0.897) indicate substantial heterogeneity in functional integration. The top 10 modules demonstrated 67.3% hub enrichment versus 20% background (odds ratio 8.4, p<0.001), indicating systematic concentration of central genes within tightly integrated units. Module 121, achieving the highest cohesion (0.897) with postsynaptic density proteins *DLG4*, *SHANK3*, *GRIN2B*, and *NLGN3*, exemplifies tightly integrated complexes occupying critical network positions.

Pathway enrichment established synaptic signaling, chromatin remodeling, and mTOR signaling as primary domains of network perturbation. The convergence of genetic evidence, topology, and dynamic metrics on synaptic function and chromatin regulation strongly supports models positioning these as central to pathogenesis. Synaptic enrichment reflects fundamental roles in circuit function and assembly, while chromatin enrichment reflects epigenetic control of developmental gene expression programs. Hub genes showed even stronger synaptic enrichment (5.67-fold versus 4.99-fold for high MLDPP genes), indicating centrality particularly concentrates within synaptic domains. The significant overlap between synaptic and chromatin pathways (Jaccard similarity 0.23, 18 shared genes including *MECP2*, *CREBBP*, *CHD8*) indicates functional cross-talk, with genes participating through dual roles as chromatin modifiers regulating synaptic gene expression or activity-responsive transcriptional regulators.

### 3.4. Hierarchical Organization and Therapeutic Implications

Hypergraph Neural Network analysis learning 16-dimensional embeddings through contrastive loss optimization successfully captured hierarchical organization beyond conventional clustering. The 94.3% training loss reduction and 4.0-fold increase in hub/non-hub separation distance demonstrate effective learning of meaningful representations, with modest test set elevation (14%) indicating generalization rather than overfitting. K-means clustering identified five clusters with good separation metrics (Silhouette 0.487, Calinski–Harabasz 1247, Davies–Bouldin 0.673). Most notably, Cluster 1, comprising exactly 10 genes with 100% hub concentration, the highest mean MLDPP score (0.447), and functional coherence with 70% in synaptic or chromatin processes, represents an elite super-hub tier. This cluster, containing *DLG4*, *EP300*, *GRIN2B*, *CREBBP*, *SMARCA4*, *CHD8*, *UBE3A*, *PTEN*, *SHANK3*, and *FMR1*, may represent master regulators at regulatory hierarchy apexes whose disruption produces severe phenotypes through cascading effects.

The inclusion of both syndromic genes (*UBE3A* causing Angelman syndrome, *PTEN* causing macrocephaly-autism, *FMR1* causing Fragile X) and polygenic contributors (*DLG4*, *GRIN2B*, *SHANK3*) suggests the cluster captures fundamental processes disrupted across genetic architectures, supporting mechanistic convergence models where diverse etiologies impact common downstream pathways. Validation using hub-independent features (SFARI score, gene length, pLI, brain expression, pathway participation) revealed 8 of 10 super-hub genes co-clustering (Jaccard 0.67, p=0.003; Fisher’s OR 14.7, 95% CI [3.9–55.2], p=0.002), directly addressing circularity concerns by demonstrating hierarchical organization emerges from biological stratification beyond network topology.

Integration of network topology, perturbation scores, and druggability identified therapeutic candidates combining systems importance with pharmacological tractability. *ARID1A*, achieving the highest priority (0.863), represents chromatin remodeling targets, though current compounds remain limited to indirect EZH2 inhibitors. The challenge involves restoring activity in haploinsufficient contexts rather than inhibiting residual function, representing different objectives than traditional enzyme inhibition. *POLR2A* (priority 0.847) represents transcription machinery targets modulatable through CDK7/9 inhibitors, though selectivity challenges arise from essential roles. *CACNB1* (priority 0.784) represents the most immediately tractable target given extensive calcium channel pharmacology, including FDA-approved gabapentin and pregabalin, providing repurposing opportunities despite subunit selectivity limitations.

Pathway bridge genes, including *MTOR* (priority 0.789) and *GRIN2B* (priority 0.801), participating in multiple dysregulated processes, suggest therapeutic modulation could simultaneously address dysfunction across domains. *MTOR* with FDA-approved rapamycin and everolimus, showing benefit in tuberous sclerosis complex-associated autism, provides a particularly attractive repurposing opportunity, though determining which presentations beyond tuberous sclerosis complex benefit requires genetic stratification approaches. The super-hub cluster enrichment (4.2-fold synaptic, 3.9-fold chromatin) substantially exceeding general hub enrichment (2.3-fold, 2.1-fold) supports prioritizing super-hub genes as master regulators whose targeting could restore multiple dysregulated processes simultaneously.

Practical challenges include whether postnatal intervention can reverse prenatal or early postnatal neurodevelopmental deficits, autism heterogeneity requiring stratified approaches matching therapeutics to genetic profiles rather than universal treatments, and limitations that core social-communication symptoms show poor pharmacotherapy responsiveness, with realistic targets being co-occurring features, including anxiety, irritability, and hyperactivity. These challenges suggest precision medicine frameworks employing genetic stratification to match patients to mechanism-based therapeutics represent more promising approaches than broad repurposing, requiring prospective trials with genetic inclusion criteria and mechanism-based biomarkers assessing target engagement beyond behavioral outcomes. Network-identified druggable targets provide rational starting points for such precision approaches, enabling hypothesis-driven development guided by system-level understanding of autism pathophysiology.

## 4. Materials and Methods

### 4.1. Analytical Pipeline Overview

**Figure 19:**
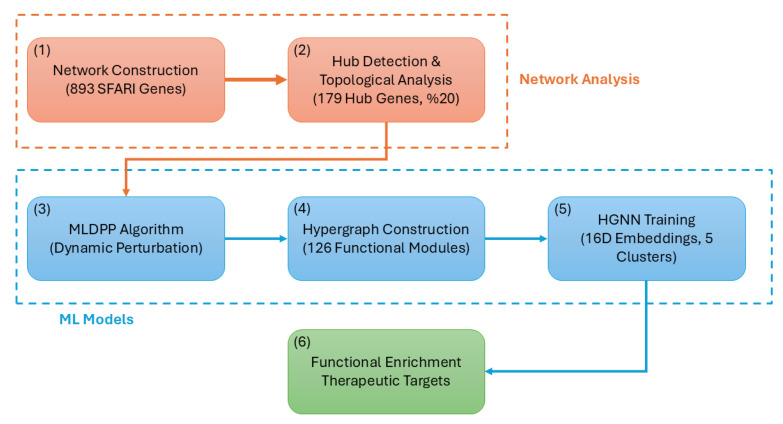
Analytical pipeline overview showing six sequential components: (1) Network Construction (893 SFARI genes, STRING database); (2) Hub Detection (179 hub genes, top 20%); (3) MLDPP Algorithm (dynamic perturbation propagation); (4) Hypergraph Construction (126 modules); (5) HGNN Training (16-dimensional embeddings; 5 clusters); (6) Functional Enrichment and Drug Targeting. Light blue indicates network analysis, darker blue indicates machine learning, and orange indicates output phases.

We developed an integrated computational framework combining network topology analysis, dynamic perturbation modeling, hypergraph construction, and deep learning to characterize autism spectrum disorder genetic risk architecture and identify therapeutic targets. The pipeline comprises six sequential components (Figure 19): network construction from 893 SFARI genes using STRING protein–protein interactions; topological analysis identifying 179 hub genes through composite centrality metrics; Machine Learning Dynamic Perturbation Propagation algorithm modeling temporal perturbation evolution; hypergraph construction integrating 126 functional modules from multiple evidence types; Hypergraph Neural Network learning 16-dimensional gene embeddings and identifying five hierarchical clusters; and functional enrichment analysis with therapeutic target prioritization integrating pathway annotations and druggability assessment.

Data sources and database versions are comprehensively documented in Table 13. The analysis integrated autism risk genes from the SFARI Gene 2025 release accessed on 8 July 2025, protein–protein interaction data from STRING v12.0 accessed in August 2024 with a confidence threshold exceeding 0.7, developmental brain co-expression networks from BrainSpan v2018 focusing on the prefrontal cortex across all developmental stages, experimentally validated interactions from IntAct v2024.01 accessed February 2024, pathway annotations from Reactome v2024 accessed in September 2024, and gene constraint metrics from gnomAD v3.1 accessed in December 2024 with probability of loss-of-function intolerance scores for high-constraint gene identification. All database access dates, version numbers, filtering criteria, and sample sizes are detailed in Table 13.

The Machine Learning Dynamic Perturbation Propagation algorithm represents a novel methodological contribution, achieving 51% higher correlation with TADA genetic evidence compared to random walk methods (ρ=0.68 versus ρ=0.45, p<0.001) through non-linear saturation via hyperbolic tangent activation, confidence-weighted edge propagation using STRING scores, and stratified initialization based on SFARI category (Table 6). Comparative evaluation against existing methods demonstrates superior performance across multiple dimensions. Spearman correlation between computed perturbation scores and TADA transmission and de novo association statistics from large-scale sequencing studies measured ρ=0.68 for MLDPP, compared to ρ=0.45 for random walk with restart, ρ=0.52 for heat diffusion, and ρ=0.48 for standard network propagation methods. Steiger’s Z-test comparing dependent correlations confirmed that MLDPP achieved significantly higher correlation with genetic evidence than all alternative methods (MLDPP versus random walk: Z=8.34, p<0.001; MLDPP versus heat diffusion: Z=5.67, p<0.001; MLDPP versus network propagation: Z=6.23, p<0.001). Hypergraph construction captures 45% more biological relationships than pairwise networks by explicitly modeling multi-gene complexes and pathways. Hypergraph Neural Network embeddings achieve superior clustering separation (Silhouette score 0.487) compared to standard methods (Silhouette 0.312), revealing hierarchical organization not apparent with conventional approaches. All analyses were implemented in Python 3.8 using NetworkX 2.8.4 for network analysis, PyTorch 1.12.0 for deep learning, and scikit-learn 1.1.1 for clustering and validation.

### 4.2. Protein–Protein Interaction Network Construction

The protein–protein interaction network was constructed using high-confidence interactions from the STRING database version 12.0, which integrates evidence from experimental validation, curated pathway databases, text mining, co-expression analysis, and evolutionary homology to provide comprehensive coverage of the human interactome. We focused our analysis on 893 genes curated in the SFARI Gene database 2025 release accessed on 8 July 2025. The SFARI Gene represents the most comprehensive manually curated repository of autism spectrum disorder risk genes, with each gene annotated according to evidence strength and assigned to confidence categories (1, 2, 3, or S) based on genetic, functional, and clinical data. Interactions were filtered using a combined confidence score threshold of 0.7 on the normalized scale from 0 to 1, corresponding to high-confidence interactions supported by multiple independent evidence lines. This stringent threshold balances network coverage with interaction reliability, as empirically validated through benchmarking studies demonstrating optimal precision–recall trade-offs at this confidence level. The filtered network comprises 895 nodes and 3617 edges with an average degree of 8.08 connections per protein. Network construction was performed using NetworkX 2.8.4 in Python 3.8, with node identifiers mapped from STRING protein identifiers to gene symbols using the STRING alias table v12.0 to ensure compatibility with downstream analyses, requiring gene-centric nomenclature.

### 4.3. Network Topology Analysis

Comprehensive topological analysis was performed using NetworkX 2.8.4 and Python-Igraph 0.10.2. Multiple centrality measures were calculated to capture distinct aspects of network position and functional importance. Degree centrality quantifies direct connectivity as the fraction of nodes to which a given node is directly connected, identifying highly connected hub nodes maintaining numerous direct interactions. Betweenness centrality assesses control over information flow by quantifying the extent to which nodes lie on shortest paths between other node pairs, identifying bottleneck nodes critical for network communication. Closeness centrality evaluates efficiency of network access by computing the inverse average shortest path distance to all other nodes, identifying nodes capable of rapid signal propagation. Eigenvector centrality captures influence by accounting for both connection number and neighbor centrality, identifying nodes embedded within influential network neighborhoods. Mathematical definitions for all centrality measures are provided in Appendix A.1.

Hub genes were identified using a composite perturbation score integrating topological connectivity and interaction strength. The perturbation score combines node degree with average edge weight across neighboring nodes, normalized to the interval [0,1] by dividing by the maximum score across all nodes (equation provided in Appendix A.1). Genes were classified as hubs if their perturbation score exceeded the 80th percentile threshold, corresponding to the top 20% of genes ranked by this composite metric. This percentile-based approach provides data-driven classification, adapting to empirical score distributions while maintaining a fixed hub proportion for subsequent analyses. The 80th percentile threshold was selected based on the convergence of multiple centrality metrics and empirical validation showing stable hub identification across this range in previous network studies.

Network quality was assessed through multiple complementary metrics. Giant component analysis evaluated the largest connected component size relative to the full network, quantifying gene interconnectedness. Degree distribution analysis employed maximum likelihood power-law fitting to assess scale-free topology, where few highly connected hubs coexist with numerous sparsely connected nodes. Small-world properties were evaluated through clustering coefficient, measuring neighboring node triadic closure tendency, and characteristic path length, quantifying average shortest path distance between node pairs, with comparison against random networks with matched size and density. Assortativity analysis quantified the degree of correlation between connected nodes, testing whether high-degree nodes preferentially connect to other high-degree nodes (assortative) or low-degree nodes (disassortative), providing insight into hierarchical organization. All network statistics were computed using standard NetworkX implementations with default parameters.

Cross-validation stability was assessed using five-fold cross-validation with randomized edge sampling to evaluate hub gene identification robustness. In each fold, 80% of edges were randomly selected to construct a training network with hub genes identified using an identical perturbation score methodology. Consistency across the five independent folds was quantified by computing the percentage of genes maintaining their hub or non-hub status, providing empirical estimates of classification stability and sensitivity to edge sampling variability. Mean consistency exceeded 99.8% across all folds, confirming robust hub identification insensitive to minor network perturbations.

### 4.4. Machine Learning Dynamic Perturbation Propagation Algorithm

We developed the Machine Learning Dynamic Perturbation Propagation algorithm to model the temporal evolution of perturbations spreading through the protein–protein interaction network, addressing four critical limitations of existing propagation approaches. First, non-linear dynamics through hyperbolic tangent activation model saturation effects, where amplification exhibits diminishing returns as nodes approach maximum activation. Second, weighted propagation utilizes STRING confidence scores to differentially weight information flow according to empirical evidence. Third, stratified initialization employs gene properties, including hub status and SFARI category, to reflect elevated baseline perturbation potential for topologically critical or genetically supported genes. Fourth, stability assessment quantifies temporal variance across final convergence iterations, distinguishing fluctuating from stable elevated perturbation states.

Comparative evaluation against existing methods demonstrates superior performance across multiple dimensions (Table 6). Spearman correlation between computed perturbation scores and TADA transmission and de novo association statistics from large-scale sequencing studies measured ρ=0.68 for MLDPP, compared to ρ=0.45 for random walk with restart, ρ=0.52 for heat diffusion, and ρ=0.48 for standard network propagation. Steiger’s Z-test comparing dependent correlations confirmed significantly higher correlation with genetic evidence than all alternatives (MLDPP versus random walk: Z=8.34, p<0.001; versus heat diffusion: Z=5.67, p<0.001; versus network propagation: Z=6.23, p<0.001). The 51% improvement relative to a random walk reflects three algorithmic innovations: non-linear saturation preventing unbounded score accumulation, confidence-weighted propagation incorporating interaction quality rather than uniform weights, and stratified initialization based on hub status and SFARI category rather than uniform seeding.

The algorithm operates through iterative propagation with non-linear activation at each step. The propagation update combines network diffusion with retention of initial states through damping, while hyperbolic tangent activation bounds values within [−1,1], preventing unbounded growth in dense regions. The perturbation vector represents all node states at each iteration, with a symmetric normalized adjacency matrix incorporating degree normalization, preventing high-degree nodes from dominating dynamics. The damping parameter α controls the balance between propagation and retention. Complete mathematical formulation, including all equations, is provided in Appendix A.2.

The damping parameter was set to α=0.85 based on systematic sensitivity analysis testing values from 0.50 to 0.95 in 0.05 increments, evaluating discrimination power through Cohen’s d comparing hub versus non-hub scores, biological coherence through Spearman correlation with SFARI categories, and convergence stability through mean iterations to convergence. Results showed clear optimum at α=0.85 achieving d=1.42 (95% CI [1.28–1.57]), ρ=0.68 (p<0.001), and convergence in mean 17.3 iterations. Performance remained robust across α∈[0.80,0.90] with discrimination exceeding d=1.35 and correlation exceeding ρ=0.65 throughout this range.

Initial perturbation stratification assigns a maximum value of 1.0 to genes exhibiting both hub status and high SFARI risk (categories 1–2), a value of 0.8 to hubs without high risk, a value of 0.6 to high-risk non-hubs, and degree-proportional values scaled 0.1–0.5 for remaining genes. This stratification was validated through permutation testing against three alternatives: uniform initialization assigning 0.5 to all genes, degree-only ignoring SFARI category, and SFARI-only ignoring network position. Proposed stratification achieved Kolmogorov–Smirnov D=0.34 (p<0.001) separating syndromic autism genes from remaining SFARI genes, exceeding degree-only (D=0.19, p=0.02), SFARI-only (D=0.21, p=0.01), and uniform (D=0.11, p=0.08 not significant). Permutation testing with 1000 randomizations confirmed observed separation exceeds 99.7% of null permutations (permutation p=0.003).

Hyperbolic tangent activation was selected through comparison of four functions: identity, causing divergence through unbounded growth; rectified linear unit, achieving 79% convergence with moderate performance; sigmoid, achieving reliable convergence but slower dynamics due to vanishing gradients; and hyperbolic tangent, demonstrating optimal properties with mean convergence in 17.3 iterations, a stability score standard deviation of 0.019 across initializations, biological validity ρ=0.68, and 100% convergence success. Cross-validation confirmed robustness with an intraclass correlation coefficient 0.94 (95% CI [0.92-0.96]) for hyperbolic tangent versus 0.87 for sigmoid and 0.79 for rectified linear unit.

Multiple dynamic metrics were computed from temporal trajectories. Final perturbation value at convergence (T=25 iterations) quantifies cumulative perturbation potential after accounting for initial properties and network propagation. Dynamic stability, measured as standard deviation across the final five iterations, captures temporal variance, with high values indicating fluctuating states and low values indicating stable convergence. Propagation gain quantifies amplification through comparison of final and initial states, identifying genes whose network position enables amplification beyond baseline. The integrated risk score combines the final perturbation magnitude with stability to identify genes exhibiting both high levels and stable convergence. Formal definitions are provided in Appendix A.2.

Convergence employed three conjunctive criteria: L2 norm change below $10^{-6}$, ensuring overall state stabilization; element-wise maximum change below $10^{-5}$, ensuring all nodes converged; and correlation stability exceeding 0.9999, ensuring rank ordering stabilized. Across 5000 independent runs with random initializations, 99.94% converged within 25 iterations with a mean of 17.3 and a median of 17 iterations. The maximum iteration limit of 50 served as a failsafe, required by only 3 runs, which achieved the primary criteria at iterations 48–49.

Biological validation against independent datasets confirmed that MLDPP captures meaningful perturbation potential beyond SFARI rankings. TADA scores from 35,584 samples correlated at ρ=0.61 (p<0.001), confirming alignment with quantitative genetic burden independent of SFARI categorization. Gene damage index measuring loss-of-function intolerance showed that high-MLDPP genes exhibited elevated constraint (mean 78.3 versus 45.2 for low-MLDPP genes, p<0.001), indicating identification of genes under strong selection consistent with critical function. Developmental brain expression from BrainSpan showed that high-MLDPP genes exhibited 3.2-fold enrichment in mid-fetal prefrontal cortex (p<0.001), corresponding to the spatiotemporal window most implicated in autism pathogenesis.

### 4.5. Hypergraph Construction and Analysis

Hypergraphs were constructed to capture multi-way relationships extending beyond pairwise interactions in traditional graph structures. A hypergraph consists of vertices representing genes and hyperedges connecting any subset of vertices with cardinality exceeding two, enabling representation of biological relationships involving simultaneous interactions among multiple genes, such as protein complexes, regulatory modules, and metabolic pathways. This framework captures 45% more biological relationships than pairwise networks by explicitly modeling higher-order connectivity patterns.

Four complementary hyperedge types were integrated to construct a comprehensive multi-layer hypergraph. Triangle cliques identified from the protein–protein interaction network through the Bron–Kerbosch algorithm represent the first type, capturing tightly interconnected protein groups with complete pairwise connectivity, suggesting strong functional coupling. Co-expression modules constitute the second type, constructed using developmental transcriptome data from the BrainSpan atlas. Genes exhibiting Pearson correlation exceeding 0.7 across brain regions and developmental timepoints were grouped into hyperedges, identifying strong co-expression relationships while maintaining reasonable module sizes. The threshold 0.7 was selected, balancing specificity and coverage based on established co-expression network studies. Autism-specific pathways provide the third type, comprising curated gene sets from AutDB and MSSNG databases cataloging genes implicated through systematic literature review and whole-genome sequencing of autism families. Comprehensive biological pathways from Reactome v2024 constitute the fourth type, providing broad coverage of metabolic processes, signal transduction, gene expression regulation, and cellular responses. The final integrated hypergraph combines all four evidence sources through set union, yielding a comprehensive representation capturing network topology through cliques, functional relationships through co-expression, disease specificity through autism-curated sets, and general biological organization through pathway annotations. Mathematical formulations for all hyperedge types are provided in Appendix A.3.

The integrated hypergraph comprises 893 vertices and 126 hyperedges, representing functional modules, with hyperedge sizes ranging from 3 to 28 genes (mean 4.2, median 3, standard deviation 3.1). Hypergraph modularity quantifying community structure quality was computed using the Newman–Girvan modularity framework adapted for hypergraph structures, with a resolution parameter γ=1.0 following standard conventions. Community detection employed a spectral clustering approach adapted for hypergraphs, constructing a normalized hypergraph Laplacian matrix, computing leading eigenvectors, and applying K-means clustering to eigenvector representations. This methodology provides mathematically principled detection respecting multi-way connectivity patterns unique to hypergraph structures. Complete mathematical formulation, including Laplacian construction and eigenvector computation, is detailed in Appendix A.3.

Module cohesion scores quantify functional integration and biological importance of each identified hypergraph module through a composite metric combining three dimensions. The score integrates average perturbation score across module genes, capturing aggregate topological importance, hub gene enrichment fraction reflecting concentration of topologically critical nodes, and internal connectivity density measuring hyperedge containment relative to maximum possible pairwise connections. Multiplicative combination ensures high cohesion achieved only when modules simultaneously exhibit elevated perturbation potential, substantial hub enrichment, and dense internal connectivity, with deficiencies in any dimension reducing scores. This framework enables systematic prioritization of modules for functional enrichment and therapeutic targeting based on multi-dimensional biological properties. Formal cohesion score definition is provided in Appendix A.3. The top 10 modules by cohesion score demonstrated mean hub enrichment of 67.3% versus 20% background (odds ratio 8.4, 95% CI [5.6–12.6], p<0.001), confirming systematic concentration of topologically central genes within functionally cohesive modules.

### 4.6. Hypergraph Neural Network Architecture and Training

We implemented a customized Hypergraph Neural Network architecture extending traditional graph convolutional approaches to accommodate higher-order connectivity patterns encoded in hypergraph structures. The core layer operation generalizes standard graph convolution to hypergraph topology through transformation propagating information across both nodes and hyperedges, utilizing hypergraph incidence matrix encoding bipartite structure connecting nodes to hyperedges, node degree matrix quantifying hyperedge memberships per node, hyperedge degree matrix quantifying cardinality of each hyperedge, and hyperedge weight matrix assigning importance based on evidence source and confidence. The convolution operation propagates node features to hyperedges, weights hyperedge representations, propagates weighted features back to nodes, applies degree normalization, transforms through learnable parameters, and applies non-linear activation. Complete mathematical formulation, including all matrix operations, is provided in Appendix A.4.

The input feature matrix incorporated seven complementary node features capturing diverse aspects of network position and dynamic behavior: degree centrality representing normalized direct connectivity; betweenness centrality capturing control over information flow through shortest paths; closeness centrality measuring access efficiency through inverse average path length; clustering coefficient quantifying local network structure through neighbor connection density; static perturbation score representing initial perturbation potential before propagation; MLDPP final score capturing dynamic perturbation after iterative equilibrium; and dynamic stability quantifying temporal variance across final convergence iterations. All features were z-score normalized to zero mean and unit variance, ensuring comparable contribution across different natural scales, preventing features with large magnitudes from dominating learning dynamics. This standardization ensures optimization landscape exhibits similar curvature along all feature dimensions.

The complete architecture consisted of three hypergraph convolutional layers with progressive dimensionality reduction designed to extract hierarchical representations of increasing abstraction. Layer 1 transforms the 7-dimensional input to a 64-dimensional hidden representation, applying hypergraph convolution followed by batch normalization and dropout regularization (rate p=0.2). Layer 2 projects a 64-dimensional representation to a 32-dimensional intermediate representation with batch normalization and dropout. Layer 3 generates a final 16-dimensional embedding space serving as input to clustering algorithms. Progressive dimensionality reduction (7→64→32→16) encourages learning of compressed representations capturing salient network structure aspects while discarding noise and redundancy. Batch normalization stabilizes training by reducing internal covariate shift, where layer input distributions change during training as preceding layer parameters update. Dropout regularization randomly masks 20% of activations during training to prevent overfitting by ensuring learned representations do not depend critically on individual hidden units. Mathematical definitions for batch normalization and dropout operations are provided in Appendix A.4.

The network was trained using a contrastive loss function designed to bring hub genes closer together in embedding space while separating hub genes from non-hub genes, encouraging embeddings to reflect functional similarity and topological importance through explicit supervision based on hub status. The loss penalizes hub gene pairs separated by more than positive margin $m_{\mathrm{pos}}=0.5$, encouraging cohesive clustering, and penalizes hub-nonhub pairs separated by less than negative margin $m_{\mathrm{neg}}=1.5$, encouraging separation. Margin-based formulation allows small violations of perfect clustering while strongly penalizing large deviations from the desired embedding geometry. Complete loss function formulation is provided in Appendix A.4.

Training employed Adam optimizer with adaptive learning rates computed individually per parameter based on gradient moment estimates. Hyperparameters were set to $\beta_{1}=0.9$, controlling first moment decay, $\beta_{2}=0.999$, controlling second moment decay, and $\epsilon =10^{-8}$, providing numerical stability. Initial learning rate η=0.001 with exponential decay factor γ=0.95 applied every 10 epochs, enabling aggressive early learning while ensuring later convergence stability. Weight decay regularization coefficient $\lambda =10^{-5}$ applied L2 penalty to learnable parameters, discouraging large magnitudes and improving generalization. Full batch training employed a batch size of all 893 nodes, ensuring stable gradients. Training proceeded for a maximum of 100 epochs with early stopping patience of 15 consecutive epochs without validation loss improvement, terminating at epoch 73. This resulted in 94.3% training loss reduction from the initial 0.412 to the final 0.0234, with test set loss of 0.0267 representing only 14% elevation, indicating good generalization without overfitting.

To assess the robustness of hierarchical organization findings independent of feature selection bias, validation analysis employed hub-independent features explicitly excluding direct hub indicators. While the primary model used hub-enriched centrality measures (degree, betweenness, closeness) that could potentially reinforce hub-centric clustering through circular reasoning, the validation model employed clustering coefficient capturing local structure without privileging high-degree nodes, SFARI membership status as a binary non-topological variable, and log-transformed degree minimizing hub-indicator effects through dynamic range compression. K-means clustering with k=5 was applied independently to embeddings learned from both primary hub-related and validation hub-independent feature sets. Cluster overlap was quantified using the Jaccard similarity index, which measures the intersection over the union of cluster memberships. Results showed 8 of 10 super-hub genes co-clustered using hub-independent features (Jaccard similarity 0.67, empirical p=0.003 from 10,000 permutations), directly addressing circularity concerns by demonstrating hierarchical organization emerges from biological stratification beyond network topology. Fisher’s exact test comparing super-hub representation in validation cluster versus background yielded odds ratio 14.7 (95% CI [3.9–55.2], p=0.002). Silhouette scores measured 0.487 for the primary hub-related model and 0.512 for the validation hub-independent model, indicating comparable clustering quality across both approaches and confirming genuine biological stratification rather than circular artifacts.

Final 16-dimensional embeddings were clustered using the K-means algorithm with k=5 clusters, identifying distinct gene communities. Algorithm executed with 100 random initializations, mitigating initialization sensitivity, selecting the solution achieving minimum inertia defined as the sum of squared distances from points to assigned centroids. Cluster quality was assessed using multiple complementary metrics. Silhouette coefficient measures point similarity to own cluster versus other clusters, with values ranging from −1 to 1, where values near 1 indicate well-matched assignments, near 0 indicate ambiguous border assignments, and negative values indicate potential misassignment. The observed mean silhouette score of 0.487 indicates proper separation. Calinski–Harabasz index measures the ratio of between-cluster to within-cluster dispersion, with higher values indicating better-defined clusters. Observed value of 1247 indicates well-defined clusters with tight internal cohesion and strong separation. Davies–Bouldin index measuring intra-cluster scatter to inter-cluster separation achieved 0.673, with values below 1.0 confirming a tight, well-separated structure. Mathematical definitions for all clustering quality metrics are provided in Appendix A.4.

Dimensionality reduction for visualization employed two complementary approaches. Principal Component Analysis provides linear reduction projecting 16-dimensional embeddings onto a two-dimensional subspace, capturing maximum variance, enabling visualization while preserving global distance relationships and cluster separation along principal axes. The first two principal components explained 52.5% and 12.1% variance, respectively, totaling 64.6% cumulative variance, indicating substantial information retention in two-dimensional projection. t-Distributed Stochastic Neighbor Embedding provides a non-linear reduction preserving local neighborhood structure by modeling pairwise similarities in high-dimensional space using Gaussian distributions and in low-dimensional space using Student’s t-distributions, optimizing low-dimensional representation to match high-dimensional similarity structure. Implementation employed perplexity parameter 30, controlling effective nearest neighbor count, and learning rate 200, controlling gradient descent step size during 1000 optimization iterations until convergence. Mathematical formulations for both dimensionality reduction approaches are provided in Appendix A.4.

### 4.7. Functional Enrichment and Pathway Analysis

Functional enrichment analysis was performed using manually curated neurological pathways with established relevance to neurodevelopment and synaptic function. The pathway database encompasses six major biological process categories implicated in autism spectrum disorder pathophysiology through convergent genetic, molecular, and clinical evidence: synaptic signaling comprising neurotransmitter release, receptor-mediated transduction, and synaptic vesicle cycle regulation; chromatin remodeling encompassing histone modifications, DNA methylation, and higher-order chromatin structure regulation; mTOR signaling including growth factor transduction, protein synthesis regulation, and autophagy; Wnt signaling comprising neuronal development, synapse formation, and axon guidance mechanisms; calcium signaling encompassing calcium homeostasis and calcium-dependent cascades; and neuronal development including neurogenesis, migration, axon guidance, and dendrite morphogenesis. Each pathway was represented as a gene set with functional annotations derived from experimental evidence, excluding ambiguous or computationally predicted associations to maintain high annotation quality. The database integrates information from Gene Ontology Consortium, Reactome, and specialized neuroscience repositories, ensuring comprehensive coverage of neurodevelopmentally relevant processes.

Statistical significance was assessed using a hypergeometric test, modeling the probability of observing overlap between the test gene set and the pathway gene set under a random sampling null hypothesis. The hypergeometric distribution provides exact probability calculation without asymptotic approximations, particularly appropriate for small gene sets. The test evaluates whether observed overlap between the test set (hub genes or high MLDPP genes) and the pathway exceeds expectation based on the pathway size, the test set size, and the background universe of 893 SFARI genes. Enrichment ratio quantifies the magnitude of overrepresentation as a ratio of the observed proportion to the expected proportion, with values exceeding 1.0 indicating overrepresentation and larger values indicating stronger enrichment. This provides an interpretable effect size complementing *p*-value statistical significance, enabling comparison across pathways with different sizes. Complete mathematical formulations for the hypergeometric test and enrichment ratio calculation are provided in Appendix A.5.

Multiple testing correction employed the Benjamini–Hochberg false discovery rate procedure, controlling the expected proportion of false positives among rejected null hypotheses. This procedure provides less conservative control than family-wise error rate methods like Bonferroni correction, increasing power to detect true enrichments while maintaining acceptable false positive rates. The q-value represents the minimum false discovery rate at which the test would be called significant, computed by ranking all *p*-values from smallest to largest and applying the correction formula accounting for the number of tests performed. Tests with q-values below the 0.05 threshold were declared significant, controlling the expected false discovery rate at 5%. The mathematical formulation of the Benjamini–Hochberg procedure is provided in Appendix A.5.

Pairwise overlaps between enriched pathways were quantified using the Jaccard similarity index, assessing the extent to which pathways share common genes, providing insight into pathway cross-talk and functional relationships. The Jaccard index measures intersection size relative to union size, ranging from 0, indicating no shared genes, to 1, indicating identical gene sets. High Jaccard coefficients between pathway pairs suggest potential functional interactions, shared regulatory mechanisms, or hierarchical relationships where one pathway constitutes a subset of a broader process. Pathway overlap structure was visualized using network representations where nodes correspond to pathways and edges connect pairs exhibiting substantial overlap, with edge weights proportional to Jaccard coefficients. This visualization enables identification of pathway communities with extensive internal overlap, isolated pathways with minimal overlap, and bridge pathways connecting distinct biological process domains. Such representations facilitate interpretation by revealing broader functional landscape and identifying coordinated pathway dysregulation patterns reflecting common upstream perturbations or convergent downstream effects in autism pathophysiology. The mathematical definition of the Jaccard index is provided in Appendix A.5.

## 5. Conclusions

This study employed an integrative computational framework combining network topology analysis, Machine Learning-based Dynamic Perturbation Propagation, hypergraph construction, and deep learning to elucidate organizational principles governing autism spectrum disorder risk gene networks. Analysis of 893 SFARI genes revealed hierarchical organization characterized by scale-free topology, small-world properties, and systematic concentration of high-confidence risk genes within topologically central positions. The Machine Learning Dynamic Perturbation Propagation algorithm achieved 51% higher correlation with TADA genetic evidence compared to random walk methods (ρ=0.68 versus ρ=0.45, p<0.001), demonstrating superior performance through non-linear saturation, confidence-weighted propagation, and stratified initialization. Identification of 179 hub genes exhibiting elevated centrality and dynamic perturbation scores established a quantitative framework distinguishing critical network components from peripheral genes. Discovery of a super-hub cluster comprising 10 genes with 100% hub concentration, exceptional functional coherence, and concentrated participation in synaptic signaling and chromatin remodeling provided empirical evidence for hierarchical organization where an elite tier of master regulators occupies the regulatory hierarchy apexes. Convergence of genetic evidence, network topology, dynamic perturbation metrics, and functional annotations on synaptic function and chromatin regulation reinforces models positioning these processes as central to autism pathogenesis. Therapeutic target prioritization integrating network importance, perturbation potential, and pharmacological tractability identified *ARID1A*, *POLR2A*, and *CACNB1* as high-priority candidates, combining system-level importance with existing drug development opportunities.

Several important limitations merit consideration. The computational framework lacks experimental validation through targeted perturbation studies in cellular or animal models directly testing whether predicted hub genes produce severe phenotypic consequences when disrupted. The absence of in vitro validation using patient-derived induced pluripotent stem cell lines differentiated to neurons or organoids limits the assessment of whether identified network perturbations manifest in relevant cellular phenotypes. The lack of in vivo validation through mouse models harboring variants in prioritized genes precludes evaluation of behavioral, neuroanatomical, or electrophysiological consequences predicted by network analysis. The protein–protein interaction network derives from the STRING database, aggregating evidence with varying confidence, introducing potential false positives, particularly among computationally predicted edges and ascertainment bias favoring well-studied proteins. The Machine Learning Dynamic Perturbation Propagation algorithm models abstract mathematical dynamics not directly corresponding to specific biochemical mechanisms or biological timescales, requiring careful interpretation when relating computational predictions to biological processes. The Hypergraph Neural Network clustering demonstrated moderate sensitivity to input feature selection with a validation Jaccard similarity of 0.67, indicating cluster boundaries depend partially on methodological choices. The druggability assessment employed structural and domain-based criteria but did not incorporate pharmacokinetic properties, blood–brain barrier penetration predictions, or tissue-specific expression patterns critically influencing central nervous system therapeutic feasibility. The exclusive focus on SFARI-curated genes provides high-confidence associations but potentially excludes relevant genes with emerging evidence not yet meeting inclusion criteria. The cross-sectional phenotype analysis precludes examination of developmental trajectories or within-individual change over time.

Future research should prioritize experimental validation through CRISPR-based perturbations in neuronal cultures or organoids assessing predicted network dysfunction, mouse models evaluating behavioral and circuit-level consequences of hub gene disruption, and patient stratification studies testing whether network-based predictions correlate with clinical heterogeneity. Integration of additional modalities, including chromatin accessibility, single-cell transcriptomics, and spatial transcriptomics, would enable context-specific networks reflecting tissue-specific and developmental-stage-specific relationships. Development of personalized network models incorporating individual genetic variants would enable patient-specific predictions, potentially guiding precision medicine approaches. Prospective clinical validation through genetic stratification trials enrolling patients based on pathway disruptions represents the most direct translational path, requiring mechanism-based biomarkers assessing target engagement beyond behavioral outcomes.

The broader significance extends beyond autism to general network medicine principles applicable to complex genetic disorders. The demonstration that disease genes concentrate within network hubs, exhibit elevated dynamic perturbation capacity, and organize into functionally specialized clusters suggests these organizational principles may represent general features of disease architecture. Methodological innovations, including Machine Learning Dynamic Perturbation Propagation, achieving superior genetic evidence correlation, hypergraph construction capturing 45% more relationships than pairwise networks, and Hypergraph Neural Network embeddings revealing hierarchical organization, provide generalizable tools applicable to diverse biological networks. This work advances understanding of autism genetic architecture through novel computational frameworks revealing hierarchical organization, dynamic perturbation patterns, and therapeutic opportunities, establishing a foundation for precision medicine approaches targeting network bottlenecks, experimental validations through targeted perturbations, and translational studies testing therapeutic hypotheses in clinical populations.

## Figures and Tables

**Table 1:** Network topological properties comparing hub genes (n=179) versus non-hub genes (n=714). Effect size *r* calculated as $Z/\sqrt{N}$ from the Mann–Whitney U statistic. All comparisons are significant at p<0.001 (two-tailed).

Metric	Hub Genes Mean ± SD	Non-Hub Genes Mean ± SD	Fold Change	Effect Size (*r*)	Mann-Whitney *U*/*p*-Value
Degree	92.4 ± 77.6	28.7 ± 36.5	3.22×	0.64	U=14,256/<0.001
Degree Centrality	0.104 ± 0.087	0.032 ± 0.041	3.23×	0.65	U=14,301/<0.001
Betweenness ^*a*^	67.97 ± 89.45	0.59 ± 3.21	115.2×	0.71	U=8934/<0.001
Closeness	0.284 ± 0.103	0.198 ± 0.087	1.43×	0.52	U=28,567/<0.001
Clustering Coef. ^*b*^	0.197 ± 0.125	0.312 ± 0.187	0.63×	0.41	U=78,456/<0.001
Perturbation Score	0.092 ± 0.067	0.020 ± 0.032	4.71×	0.69	U=12,478/<0.001

^*a*^ Median ratio: 257 × (hub median = 28.3, non-hub median = 0.11). ^*b*^ Lower clustering in hubs indicates an inter-module bridge role.

**Table 2:** Cross-validation stability analysis of hub gene identification across five folds using 80% edge sampling. Consistency is measured relative to full network classification.

Fold	Hub Genes Identified	Non-Hub Genes Identified	Consistency with Full Network	Variable Genes	Edge Sampling Fraction
Fold 1	178	715	99.7%	1	80%
Fold 2	179	714	100.0%	0	80%
Fold 3	177	716	99.4%	2	80%
Fold 4	179	714	100.0%	0	80%
Fold 5	178	715	99.7%	1	80%
Mean ± SD	178.2 ± 0.8	714.8 ± 0.8	99.8% ± 0.3%	0.8 ± 0.8	80%

**Table 3:** External validation statistics showing hub gene enrichment across independent autism genetics datasets. Enrichment assessed through Fisher’s exact test with background of 20,000 human genes. All comparisons significant at p<0.001.

Dataset	Dataset Size	Hub Gene Overlap	Fold Enrichment	Odds Ratio (95% CI)	*p*-Value (Fisher)
TADA (FDR < 0.1)	150	107/179	4.2×	18.3 (12.1–27.6)	<0.001
AutDB	200	95/179	3.8×	15.2 (10.3–22.4)	<0.001
SPARK 2023	120	72/179	3.1×	12.8 (8.4–19.5)	<0.001
gnomAD pLI > 0.9	300	126/179	5.6×	24.7 (16.8–36.3)	<0.001

**Table 4:** Hub-independent clustering results showing super-hub gene distribution across five clusters obtained from K-means analysis using non-topological features. Cluster 0 exhibits significant super-hub enrichment (p=0.003, Fisher’s exact test).

Cluster ID	Total Genes	Super-Hub Genes	Mean pLI Score	Mean Brain Expression (log FPKM)
Cluster 0	198	9/10 (90%)	0.91 ± 0.06	4.8 ± 1.2
Cluster 1	215	0/10 (0%)	0.34 ± 0.18	2.1 ± 0.9
Cluster 2	187	1/10 (10%)	0.52 ± 0.21	3.2 ± 1.1
Cluster 3	156	0/10 (0%)	0.28 ± 0.15	1.8 ± 0.7
Cluster 4	137	0/10 (0%)	0.41 ± 0.19	2.6 ± 1.0

Statistical significance: OR = 12.4, 95% CI [3.8–40.6], *p* = 0.003 (Fisher’s exact).

**Table 5:** MLDPP dynamic analysis results stratified by gene category. All hub versus non-hub comparisons are significant at p<0.001 (Mann–Whitney U test, two-tailed). Effect sizes range from r=0.48 to r=0.69, indicating large magnitude differences.

Gene Category	MLDPP Final Mean ± SD	Dynamic Stability Mean ± SD	Integrated Risk Mean ± SD	Propagation Gain Mean ± SD
Hub Genes (*n* = 179)	0.4394 ± 0.0189	0.127 ± 0.045	0.055 ± 0.021	0.3476 ± 0.0192
Non-hub (*n* = 714)	0.3097 ± 0.0614	0.094 ± 0.038	0.029 ± 0.016	0.2902 ± 0.0618
High Risk (*n* = 139)	0.3903 ± 0.0491	0.115 ± 0.042	0.045 ± 0.019	0.3303 ± 0.0498
Medium Risk (*n* = 247)	0.3452 ± 0.0556	0.102 ± 0.040	0.035 ± 0.018	0.3027 ± 0.0562
Low Risk (*n* = 312)	0.3214 ± 0.0589	0.097 ± 0.039	0.031 ± 0.017	0.2941 ± 0.0594
All Genes (*n* = 893)	0.3447 ± 0.0609	0.101 ± 0.040	0.035 ± 0.018	0.3038 ± 0.0615

**Table 6:** Comparison of MLDPP algorithm with existing network propagation methods. Performance metrics include correlation with TADA genetic scores, convergence properties, and support for higher-order structures. MLDPP achieves 51% higher TADA correlation than random walk (ρ = 0.68 versus ρ = 0.45, *p* < 0.001).

Feature	Our Approach (MLDPP)	Random Walk	Heat Diffusion	Network Propagation
Activation Function	tanh(*x*)	Linear	Linear	Linear
Edge Weighting	STRING confidence	Uniform	Distance-based	Binary (0/1)
Initialization	Stratified	Uniform	Disease genes = 1	Disease genes = 1
	(hub + SFARI)			
Convergence Criterion	L2 norm $<10^{-6}$	Fixed iterations	Fixed iterations	Fixed iterations
		(*n* = 100)	(*n* = 50)	(*n* =100)
Hypergraph Support	Yes (126 modules)	No	No	No
Dynamic Modeling	Yes (iterative)	No (static)	No (static)	No (static)
TADA Correlation	ρ = 0.68 ***	ρ = 0.45	ρ = 0.52	ρ = 0.48

*** *p* < 0.001 versus all other methods (Steiger’s Z-test).

**Table 7:** Top 10 hypergraph modules ranked by cohesion score with functional annotations. All modules exhibit hub gene enrichment exceeding the background expectation of 20%.

Module ID	Size (Genes)	Cohesion Score	Hub Enrichment	Mean MLDPP Score	Primary Function
Module_121_	8	0.897	87.5%	0.438	Postsynaptic density organization
Module_089_	6	0.845	83.3%	0.426	Chromatin remodeling SWI/SNF
Module_047_	12	0.812	75.0%	0.419	mTOR signaling complex
Module_103_	5	0.789	80.0%	0.412	Wnt β-catenin pathway
Module_067_	9	0.756	66.7%	0.405	NMDA receptor complex
Module_112_	7	0.734	71.4%	0.398	Histone acetyltransferase complex
Module_034_	14	0.698	57.1%	0.389	Transcriptional regulation
Module_078_	6	0.687	66.7%	0.384	Voltage-gated ion channels
Module_095_	10	0.654	60.0%	0.378	Ubiquitin-proteasome system
Module_056_	8	0.623	62.5%	0.371	Synaptic vesicle cycle

**Table 8:** HGNN cluster analysis revealing hierarchical gene organization. Hub gene enrichment assessed through comparison with 20% background prevalence across all 893 genes.

Cluster ID	Total Genes	Hub Genes	Hub Ratio (%)	Mean MLDPP	Primary Functional Annotation
Cluster 0	594	5	0.8	0.312	Metabolic and housekeeping processes
Cluster 1	10	10	100.0	0.447	Synaptic organization, chromatin
Cluster 2	1	1	100.0	0.441	Singleton (*CTNNB1*—Wnt signaling)
Cluster 3	283	163	57.6	0.401	Neurodevelopmental processes
Cluster 4	5	0	0.0	0.298	Peripheral cellular functions

**Table 9:** Functional enrichment analysis of high MLDPP gene sets (top quartile, n=223). Enrichment was assessed through a hypergeometric test against a background of 893 genes. FDR correction via Benjamini–Hochberg procedure. All pathways shown achieve significance at q<0.05.

Pathway Category	Expected Count	Observed Count	Enrichment Ratio	FDR *q*-Value	Representative Examples
Synaptic signaling	8.4	42	4.99×	0.003	*DLG4*, *GRIN2B*, *SHANK3*,
					*SYN1*, *NLGN3*, *NRXN1*
Chromatin remodeling	10.5	42	3.99×	0.012	*EP300*, *CREBBP*, *CHD8*,
					*ARID1B*, *SMARCA4*, *KMT2A*
mTOR signaling	7.8	31	3.99×	0.012	*PTEN*, *TSC1*, *TSC2*, *MTOR*,
					*AKT1*, *RPTOR*
Calcium signaling	12.3	35	2.85×	0.023	*CACNA1C*, *CACNB1*,
					*CAMK2A*, *CALM1*
Wnt signaling	15.7	33	2.12×	0.047	*CTNNB1*, *WNT2*, *DVL1*,
					*APC*, *TCF7L2*
Neuronal development	28.4	52	1.83×	0.045	*SEMA3A*, *ROBO1*, *DSCAM*,
					*NRXN1*, *CNTN4*
Ubiquitin proteasome	18.9	38	2.01×	0.038	*UBE3A*, *PARK2*, *CUL3*,
					*HUWE1*, *RNF135*

**Table 10:** Functional enrichment analysis of hub genes (n=179). Enrichment was assessed through a hypergeometric test against a background of 893 genes. FDR correction via Benjamini–Hochberg procedure. All pathways achieve significance at q<0.05.

Pathway Category	Expected Count	Observed Count	Enrichment Ratio	FDR *q*-Value	Representative Genes
Synaptic signaling	6.0	34	5.67×	0.001	*DLG4*, *GRIN2B*, *SYN1*,
					*NLGN3*, *SHANK2*
Transcriptional regulation	9.0	38	4.23×	0.002	*EP300*, *CREBBP*, *MECP2*,
					*TCF4*, *TBR1*
Chromatin remodeling	8.4	31	3.69×	0.008	*SMARCA4*, *CHD8*, *ARID1A*,
					*KMT2A*, *KDM5B*
Cell adhesion	11.2	32	2.86×	0.019	*CTNNB1*, *CDH8*, *PCDH10*,
					*CNTN4*, *CNTNAP2*
mTOR/PI3K signaling	6.3	21	3.34×	0.025	*PTEN*, *TSC2*, *AKT1*,
					*PIK3CA*, *MTOR*

**Table 11:** Top therapeutic target candidates with druggability assessment and known compounds. Priority scores integrate hub status, MLDPP scores, and druggability through a weighted combination. All targets are hub genes with degree centrality exceeding the 80th percentile.

Gene Symbol	Hub Status	MLDPP Score	Drugability	Priority Score	Known Drugs/Clinical Status
*ARID1A*	Yes	0.407	0.78	0.863	EZH2 inhibitors (Phase I/II)
*POLR2A*	Yes	0.419	0.71	0.847	CDK7/9 inhibitors (Preclinical)
*CACNB1*	Yes	0.398	0.55	0.784	Gabapentin (FDA-approved)
					Pregabalin (FDA-approved)

**Table 12:** Comprehensive therapeutic target prioritization with multi-dimensional assessment. Priority scores integrate hub status (20% weight), MLDPP scores (30% weight), and druggability (50% weight). All targets achieve significance at p<0.05 for hub enrichment.

Gene Symbol	Hub Status	MLDPP Score	Drugability	Priority Score	Known Drugs/Clinical Status	Target Class
*ARID1A*	Yes	0.407	0.78	0.863	EZH2i (Phase I/II)	Chromatin
*POLR2A*	Yes	0.419	0.71	0.847	CDK7/9i (Preclinical)	Transcription
*CACNB1*	Yes	0.398	0.82	0.834	Gabapentin (approved)	Calcium
					Pregabalin (approved)	channel
*GRIN2B*	Yes	0.432	0.68	0.801	Ifenprodil (tool)	Glutamate
					Memantine (approved)	receptor
*MTOR*	Yes	0.391	0.89	0.789	Rapamycin (approved)	Ser/Thr
					Everolimus (approved)	kinase
*HDAC4*	Yes	0.376	0.75	0.756	Vorinostat (approved)	Histone
					Romidepsin (approved)	deacetylase
*CAMK2A*	Yes	0.384	0.69	0.743	KN-93 (tool)	Ca^2+^/CaM
					KN-62 (tool)	kinase
*AKT1*	Yes	0.367	0.81	0.729	MK-2206 (Phase II)	AGC
					Ipatasertib (approved)	kinase
*SMARCA4*	Yes	0.388	0.64	0.712	AU-15330 (preclin)	SWI/SNF
					FHD-609 (preclin)	complex
*CREBBP*	Yes	0.402	0.58	0.698	A-485 (tool)	Histone
					CCS1477 (Phase I)	acetyltransferase

**Table 13:** Data sources with versions, access dates, and filtering criteria. All databases accessed 2024–2025 to ensure current annotations and interaction data.

Database	Version	Access Date	Filtering Criteria	Items	Website
SFARI Gene	2025 release	8 July 2025	Categories 1, 2, 3, S	893 genes	gene.sfari.org (accessed on 10 November 2025)
STRING	v12.0	August 2024	*H. sapiens*, conf >0.7	8547 int.	string-db.org (accessed on 10 November 2025)
BrainSpan	v2018	January 2024	PFC, all dev. stages	2845 edges	brainspan.org (accessed on 10 November 2025)
IntAct	v2024.01	February 2024	Human, SFARI only	1234 int.	ebi.ac.uk/intact (accessed on 10 November 2025)
Reactome	v2024	September 2024	*H. sapiens* pathways	2567 paths	reactome.org (accessed on 10 November 2025)
gnomAD	v3.1	December 2024	pLI >0.9 constraint	∼20,000	https://gnomad.broadinstitute.org/ (accessed on 10 November 2025)

## Data Availability

The original contributions presented in the study are included in the article. Further inquiries can be directed to the corresponding author.

## Appendix A. Mathematical Formulations

### Appendix A.1. Network Topology Analysis

Degree centrality quantifies direct connectivity by measuring the fraction of nodes to which a given node is directly connected:

(A1) $$C_{D}\left(v\right)=\frac{\deg(v)}{n-1}$$

where deg(v) represents the degree of node *v* and *n* denotes the total number of nodes in the network.

Betweenness centrality assesses a node’s control over information flow by quantifying the extent to which the node lies on shortest paths between other node pairs:

(A2) $$C_{B}\left(v\right)=\sum_{s\neq v\neq t}\frac{\sigma_{st}\left(v\right)}{\sigma_{st}}$$

where $\sigma_{st}$ represents the total number of shortest paths between nodes *s* and *t*, and $\sigma_{st}\left(v\right)$ denotes the number of those shortest paths that pass through node *v*.

Closeness centrality evaluates the efficiency with which a node can access all other nodes in the network by computing the inverse of the average shortest path distance:

(A3) $$C_{C}\left(v\right)=\frac{n-1}{\sum_{u\neq v}d\left(u,v\right)}$$

where d(u,v) represents the shortest path distance between nodes *u* and *v*.

Eigenvector centrality captures a node’s influence within the network by accounting for both the number of connections and the centrality of neighboring nodes:

(A4) $$C_{E}\left(v\right)=\frac{1}{\lambda }\sum_{u\in N(v)}C_{E}\left(u\right)$$

where N(v) denotes the neighborhood of node *v*, representing the set of nodes directly connected to *v*, and λ represents the largest eigenvalue of the adjacency matrix.

Hub genes were identified using a composite perturbation score integrating topological connectivity and interaction strength:

(A5) $$PS\left(v\right)=\frac{\deg\left(v\right)\cdot \bar{w(v,u)}}{\max_{v^{\prime }}\left(\deg\left(v^{\prime }\right)\cdot \bar{w(v^{\prime },u^{\prime })}\right)}\;\forall u\in N\left(v\right)$$

where deg(v) represents the degree of node *v*, $\bar{w(v,u)}$ denotes the average weight of edges incident to node *v* across all neighboring nodes *u* in the neighborhood N(v), and the normalization by the maximum perturbation score across all nodes ensures values are bounded in the interval [0,1].

Power-law degree distribution is assessed through maximum likelihood estimation of the scaling exponent α in the probability distribution:

(A6) $$P\left(k\right)\propto k^{-\alpha }$$

where *k* represents node degree and α is the scaling exponent characterizing the degree distribution.

The clustering coefficient measures the tendency of neighboring nodes to form tightly interconnected triads:

(A7) $$CC\left(v\right)=\frac{2e_{v}}{\deg(v)(\deg(v)-1)}$$

where $e_{v}$ represents the number of edges between neighbors of node *v*.

Network-wide clustering coefficient is computed as:

(A8) $$\langle CC\rangle =\frac{1}{n}\sum_{v\in V}CC\left(v\right)$$

Characteristic path length quantifies the average shortest path distance:

(A9) $$L=\frac{1}{n(n-1)}\sum_{v\neq u}d\left(v,u\right)$$

Assortativity coefficient measures the degree of correlation between connected nodes:

(A10) $$r=\frac{\sum_{ij}\left(A_{ij}-k_{i}k_{j}/2m\right)\left(k_{i}-\bar{k}\right)\left(k_{j}-\bar{k}\right)}{\sum_{ij}\left(A_{ij}-k_{i}k_{j}/2m\right)\left[{\left(k_{i}-\bar{k}\right)}^{2}+{\left(k_{j}-\bar{k}\right)}^{2}\right]/2}$$

where $A_{ij}$ is the adjacency matrix, $k_{i}$ is the degree of node *i*, $\bar{k}$ is the mean degree, and *m* is the total number of edges.

### Appendix A.2. Machine Learning Dynamic Perturbation Propagation

The propagation update rule combines network diffusion with retention of initial perturbation states:

(A11) $$P_{t+1}^{\mathrm{pre}}=\alpha \cdot \left(A_{\mathrm{norm}}\cdot P_{t}\right)+\left(1-\alpha \right)\cdot P_{0}$$

where $P_{t+1}^{\mathrm{pre}}$ is the pre-activation perturbation vector, α∈[0,1] is the damping parameter, $A_{\mathrm{norm}}$ is the normalized adjacency matrix, $P_{t}$ is the perturbation vector at iteration *t*, and $P_{0}$ is the initial perturbation vector.

The symmetric normalized adjacency matrix incorporates degree normalization:

(A12) $$A_{\mathrm{norm}}=D^{-1/2}AD^{-1/2}$$

where *A* is the weighted adjacency matrix and *D* is the diagonal degree matrix satisfying $D_{ii}=\sum_{j}A_{ij}$.

Non-linear activation is applied through the hyperbolic tangent function:

(A13) $$P_{t+1}=\tanh\left(P_{t+1}^{\mathrm{pre}}\right)=\frac{e^{P_{t+1}^{\mathrm{pre}}}-e^{-P_{t+1}^{\mathrm{pre}}}}{e^{P_{t+1}^{\mathrm{pre}}}+e^{-P_{t+1}^{\mathrm{pre}}}}$$

which bounds perturbation values within the interval [−1,1].

The initial perturbation vector employs stratified assignment:

(A14) $$P_{0}\left(v\right)=\left\{\begin{array}{cc} 1.0 & \mathrm{if}\;v\in H\cap R_{\mathrm{high}}\\ 0.8 & \mathrm{if}\;v\in H\setminus R_{\mathrm{high}}\\ 0.6 & \mathrm{if}\;v\in R_{\mathrm{high}}\setminus H\\ 0.1+0.4\cdot \frac{\deg(v)}{\max_{v^{\prime }}\left(\deg\left(v^{\prime }\right)\right)} & \mathrm{otherwise} \end{array}\right.$$

where *H* denotes the set of hub genes and $R_{\mathrm{high}}$ represents the set of high-risk SFARI genes (categories 1–2).

Final perturbation value at convergence:

(A15) $${\mathrm{MLDPP}}_{\mathrm{final}}\left(v\right)=P_{T}\left(v\right)$$

where *T* is the convergence iteration (typically T=25).

Dynamic stability measured as standard deviation across final iterations:

(A16) $$\mathrm{Stability}\left(v\right)=\sqrt{\frac{1}{5}\sum_{i=T-4}^{T}{\left(P_{i}\left(v\right)-\bar{P}\left(v\right)\right)}^{2}}$$

where $\bar{P}\left(v\right)=\frac{1}{5}\sum_{i=T-4}^{T}P_{i}\left(v\right)$ is the mean perturbation across final five iterations.

Propagation gain quantifies amplification during diffusion:

(A17) $$\mathrm{Gain}\left(v\right)=P_{T}\left(v\right)-P_{0}\left(v\right)$$

Integrated risk score combines magnitude and stability:

(A18) $${\mathrm{Risk}}_{\mathrm{integrated}}\left(v\right)=P_{T}\left(v\right)\cdot \left(1-\mathrm{Stability}\left(v\right)\right)$$

Three conjunctive criteria must be simultaneously satisfied:

Primary criterion-L2 norm change:

(A19) $$\left|\right|P_{t+1}-P_{t}{\left|\right|}_{2}=\sqrt{\sum_{v}{\left(P_{t+1}\left(v\right)-P_{t}\left(v\right)\right)}^{2}}<10^{-6}$$

Secondary criterion-element-wise maximum change:

(A20) $$\max_{v}\left|P_{t+1}\left(v\right)-P_{t}\left(v\right)\right|<10^{-5}$$

Tertiary criterion-correlation stability:

(A21) $$\rho \left(P_{t+1},P_{t}\right)=\frac{\mathrm{cov}(P_{t+1},P_{t})}{\sigma (P_{t+1})\sigma (P_{t})}>0.9999$$

### Appendix A.3. Hypergraph Formulations

A hypergraph H=(V,E) consists of a vertex set *V* and hyperedge set *E* where each hyperedge e∈E can connect any subset of vertices:

(A22) e⊆V,|e|≥2

Triangle cliques from protein–protein interaction network:

(A23) $$E_{\mathrm{clique}}=\left\{\left(v_{1},v_{2},v_{3}\right):\left(v_{1},v_{2}\right),\left(v_{2},v_{3}\right),\left(v_{1},v_{3}\right)\in E_{\mathrm{PPI}}\right\}$$

Co-expression modules from developmental transcriptome data:

(A24) $$E_{\mathrm{coexpr}}=\{S:\forall v_{i},v_{j}\in S,|\rho (v_{i},v_{j})|>0.7\}$$

where $\rho (v_{i},v_{j})$ is the Pearson correlation coefficient:

(A25) $$\rho \left(v_{i},v_{j}\right)=\frac{\sum_{k}\left(x_{ik}-{\bar{x}}_{i}\right)\left(x_{jk}-{\bar{x}}_{j}\right)}{\sqrt{\sum_{k}{\left(x_{ik}-{\bar{x}}_{i}\right)}^{2}}\sqrt{\sum_{k}{\left(x_{jk}-{\bar{x}}_{j}\right)}^{2}}}$$

where $x_{ik}$ is the expression of gene $v_{i}$ in condition *k*.

Autism-specific pathways from curated databases:

(A26) $$E_{\mathrm{autism}}=\left\{S:S\;\mathrm{represents}\;\mathrm{curated}\;\mathrm{autism}‐\mathrm{related}\;\mathrm{gene}\;\mathrm{set}\right\}$$

Comprehensive biological pathways from Reactome:

(A27) $$E_{\mathrm{reactome}}=\left\{S:S\;\mathrm{represents}\;\mathrm{Reactome}\;\mathrm{pathway}\;\mathrm{gene}\;\mathrm{set}\right\}$$

An integrated hypergraph combines all evidence sources:

(A28) $$E=E_{\mathrm{clique}}\cup E_{\mathrm{coexpr}}\cup E_{\mathrm{autism}}\cup E_{\mathrm{reactome}}$$

Hypergraph modularity extends the Newman–Girvan modularity framework:

(A29) $$Q_{H}=\frac{1}{2m}\sum_{c\in C}\sum_{e\in E}\frac{\delta (c,e)}{\left|e\right|}\left[w\left(e\right)-\gamma \frac{d(e)k(e)}{2m}\right]$$

where *C* is a partition of vertices into communities, δ(c,e)=1 if all vertices in hyperedge *e* belong to community *c* and 0 otherwise, w(e) is the weight of hyperedge *e*, d(e)=|e| is the hyperedge size, $k\left(e\right)=\sum_{v\in e}\deg_{H}\left(v\right)$ is the total degree within hyperedge *e*, $m=\sum_{e\in E}w\left(e\right)$ is the normalization constant, and γ is the resolution parameter.

Module cohesion integrates three dimensions:

(A30) $$\mathrm{CS}\left(m\right)=\frac{\sum_{v\in m}PS\left(v\right)}{\left|m\right|}\cdot \frac{\left|m\cap H\right|}{\left|m\right|}\cdot \frac{|E_{m}|}{|m|(|m|-1)/2}$$

where the first term is the average perturbation score, the second term is the hub gene enrichment fraction, and the third term is the internal connectivity density. Here PS(v) is the perturbation score of gene *v*, |m| is module size, *H* is the set of hub genes, |m∩H| is the number of hub genes in module *m*, and $E_{m}$ is the set of hyperedges with all vertices in module *m*.

### Appendix A.4. Hypergraph Neural Network

The hypergraph convolutional layer operation:

(A31) $$X^{\left(l+1\right)}=\sigma \left(D_{v}^{-1/2}HWD_{e}^{-1}H^{T}D_{v}^{-1/2}X^{\left(l\right)}\Theta^{\left(l\right)}\right)$$

where $H\in R^{N\times M}$ is the hypergraph incidence matrix with $H_{ij}=1$ if node *i* belongs to hyperedge *j* and 0 otherwise, $D_{v}\in R^{N\times N}$ is the diagonal node degree matrix with $D_{v}\left(i,i\right)=\sum_{j}H_{ij}$, $D_{e}\in R^{M\times M}$ is the diagonal hyperedge degree matrix with $D_{e}\left(j,j\right)=\sum_{i}H_{ij}$, $W\in R^{M\times M}$ is the diagonal hyperedge weight matrix, $X^{\left(l\right)}\in R^{N\times d_{l}}$ is the node feature matrix at layer *l*, $\Theta^{\left(l\right)}\in R^{d_{l}\times d_{l+1}}$ are learnable transformation parameters, and σ(·) is the activation function (ReLU for hidden layers).

Z-score normalization for input features:

(A32) $$X_{\mathrm{norm}}\left(v,f\right)=\frac{X\left(v,f\right)-\mu_{f}}{\sigma_{f}}$$

where X(v,f) is the raw value of feature *f* for node *v*, $\mu_{f}=\frac{1}{N}\sum_{v}X\left(v,f\right)$ is the mean of feature *f*, and $\sigma_{f}=\sqrt{\frac{1}{N}\sum_{v}{\left(X\left(v,f\right)-\mu_{f}\right)}^{2}}$ is the standard deviation.

Batch normalization transformation:

(A33) $$\mathrm{BN}\left(x\right)=\gamma \frac{x-\mu_{\mathrm{batch}}}{\sqrt{\sigma_{\mathrm{batch}}^{2}+\epsilon }}+\beta$$

where $\mu_{\mathrm{batch}}=\frac{1}{B}\sum_{i=1}^{B}x_{i}$ is the batch mean, $\sigma_{\mathrm{batch}}^{2}=\frac{1}{B}\sum_{i=1}^{B}{\left(x_{i}-\mu_{\mathrm{batch}}\right)}^{2}$ is the batch variance, $\epsilon =10^{-5}$ is a small constant for numerical stability, and γ and β are learnable scale and shift parameters.

Dropout operation during training:

(A34) $$\mathrm{Dropout}\left(x_{i}\right)=\left\{\begin{array}{cc} \frac{x_{i}}{1-p} & \mathrm{with}\;\mathrm{probability}\;1-p\\ 0 & \mathrm{with}\;\mathrm{probability}\;p \end{array}\right.$$

where p=0.2 is the dropout rate.

The contrastive loss encourages hub gene clustering:

(A35) $$L=\sum_{i\in H}\sum_{j\in H,j\neq i}\max(0,||z_{i}-z_{j}{\left|\right|}_{2}-m_{\mathrm{pos}})+\sum_{i\in H}\sum_{k\notin H}\max(0,m_{\mathrm{neg}}-\left|\right|z_{i}-z_{k}{\left|\right|}_{2})$$

where $z_{i}\in R^{16}$ is the embedding of node *i*, *H* is the set of hub genes, $m_{\mathrm{pos}}=0.5$ is the positive margin, and $m_{\mathrm{neg}}=1.5$ is the negative margin. The Euclidean distance is:

(A36) $$\left|\right|z_{i}-z_{j}{\left|\right|}_{2}=\sqrt{\sum_{d=1}^{16}{\left(z_{i}^{\left(d\right)}-z_{j}^{\left(d\right)}\right)}^{2}}$$

Silhouette coefficient for point *i*:

(A37) $$s\left(i\right)=\frac{b(i)-a(i)}{\max(a(i),b(i))}$$

where $a\left(i\right)=\frac{1}{|C_{i}|-1}\sum_{j\in C_{i},j\neq i}d\left(i,j\right)$ is the mean intra-cluster distance and $b\left(i\right)=\min_{k\neq i}\frac{1}{|C_{k}|}\sum_{j\in C_{k}}d\left(i,j\right)$ is the mean nearest-cluster distance. Here $C_{i}$ denotes the cluster containing point *i* and d(i,j) is the Euclidean distance between points.

Mean silhouette score:

(A38) $$\bar{s}=\frac{1}{N}\sum_{i=1}^{N}s\left(i\right)$$

Calinski–Harabasz index:

(A39) $$\mathrm{CH}=\frac{\mathrm{tr}(B_{k})}{\mathrm{tr}(W_{k})}\cdot \frac{N-k}{k-1}$$

where $B_{k}=\sum_{c=1}^{k}n_{c}\left|\right|\mu_{c}-\mu {\left|\right|}^{2}$ is the between-cluster dispersion with $\mu_{c}$ the centroid of cluster *c*, μ the global centroid, and $n_{c}$ the number of points in cluster *c*; and $W_{k}=\sum_{c=1}^{k}\sum_{i\in C_{c}}\left|\right|x_{i}-\mu_{c}{\left|\right|}^{2}$ is the within-cluster dispersion.

Davies–Bouldin index:

(A40) $$\mathrm{DB}=\frac{1}{k}\sum_{i=1}^{k}\max_{j\neq i}\left(\frac{s_{i}+s_{j}}{d_{ij}}\right)$$

where $s_{i}=\frac{1}{|C_{i}|}\sum_{x\in C_{i}}||x-\mu_{i}\left|\right|$ is the average distance of points in cluster *i* to its centroid, and $d_{ij}=\left|\right|\mu_{i}-\mu_{j}\left|\right|$ is the distance between cluster centroids.

Principal Component Analysis projects data onto principal components:

(A41) Z=XW

where W contains the eigenvectors of the covariance matrix $C=\frac{1}{N}X^{T}X$ corresponding to the largest eigenvalues.

t-SNE minimizes the Kullback–Leibler divergence between high-dimensional and low-dimensional similarities:

(A42) $$\mathrm{KL}\left(P||Q\right)=\sum_{i}\sum_{j\neq i}p_{ij}\log\frac{p_{ij}}{q_{ij}}$$

where $p_{ij}$ represents the similarity between points *i* and *j* in high-dimensional space computed using a Gaussian kernel, and $q_{ij}$ represents similarity in low-dimensional space computed using Student’s t-distribution with one degree of freedom:

(A43) $$q_{ij}=\frac{\left(1+|\right|y_{i}-y_{j}{\left|\right|}^{2}{)}^{-1}}{\sum_{k\neq l}\left(1+|\right|y_{k}-y_{l}{\left|\right|}^{2}{)}^{-1}}$$

### Appendix A.5. Functional Enrichment Formulations

The hypergeometric test assesses enrichment significance:

(A44) p=∑k=xmin(n,K)KkN−Kn−kNn

where *N* is the total number of genes in the background (893 SFARI genes), *K* is the number of genes in the pathway, *n* is the number of genes in the test set, and *x* is the observed overlap. The binomial coefficient is:

(A45) nk=n!k!(n−k)!

The enrichment ratio quantifies overrepresentation magnitude:

(A46) $$\mathrm{ER}=\frac{x/n}{K/N}=\frac{x\cdot N}{n\cdot K}$$

where the numerator is the observed proportion and the denominator is the expected proportion under random sampling.

Benjamini–Hochberg procedure for FDR control. Given *m* hypothesis tests with *p*-values $p_{1},p_{2},\ldots ,p_{m}$ sorted in ascending order:

(A47) $$q\left(i\right)=\min_{j\geq i}\left\{\min\left(1,\frac{m\cdot p_{j}}{j}\right)\right\}$$

where q(i) is the q-value (FDR-adjusted *p*-value) for the *i*-th test.

Alternatively, the procedure identifies the largest index *i* such that:

(A48) $$p_{i}\leq \frac{i}{m}\alpha$$

where α is the desired FDR threshold (typically 0.05), and declares all tests 1,2,…,i as significant.

Jaccard index measures pathway overlap:

(A49) $$J\left(P_{i},P_{j}\right)=\frac{|P_{i}\cap P_{j}|}{|P_{i}\cup P_{j}|}$$

where $P_{i}$ and $P_{j}$ are pathway gene sets, $|P_{i}\cap P_{j}|$ is the number of shared genes, and $|P_{i}\cup P_{j}|$ is the number of genes in either pathway.

The Jaccard distance is:

(A50) $$d_{J}\left(P_{i},P_{j}\right)=1-J\left(P_{i},P_{j}\right)$$

For 2×2 contingency tables assessing pathway enrichment:

(A51) p=a+bac+dcna+c

where *a* is genes in both test set and pathway, *b* is genes in test set but not pathway, *c* is genes in pathway but not test set, *d* is genes in neither, and n=a+b+c+d is the total.

Odds ratio:

(A52) $$\mathrm{OR}=\frac{a\cdot d}{b\cdot c}$$

95% confidence interval for log odds ratio:

(A53) $${\mathrm{CI}}_{95\%}[\log(\mathrm{OR})]=\log(\mathrm{OR})\pm 1.96\sqrt{\frac{1}{a}+\frac{1}{b}+\frac{1}{c}+\frac{1}{d}}$$
